# Pharmacological inhibition of bromodomain and extra-terminal proteins induces an NRF-2-mediated antiviral state that is subverted by SARS-CoV-2 infection

**DOI:** 10.1371/journal.ppat.1011657

**Published:** 2023-09-25

**Authors:** Baxolele Mhlekude, Dylan Postmus, Saskia Stenzel, January Weiner, Jenny Jansen, Francisco J. Zapatero-Belinchón, Ruth Olmer, Anja Richter, Julian Heinze, Nicolas Heinemann, Barbara Mühlemann, Simon Schroeder, Terry C. Jones, Marcel A. Müller, Christian Drosten, Andreas Pich, Volker Thiel, Ulrich Martin, Daniela Niemeyer, Gisa Gerold, Dieter Beule, Christine Goffinet

**Affiliations:** 1 Institute of Virology, Campus Charité Mitte, Charité—Universitätsmedizin Berlin, corporate member of Freie Universität Berlin and Humboldt-Universität zu Berlin, Berlin, Germany; 2 Berlin Institute of Health at Charité–Universitätsmedizin Berlin, Berlin, Germany; 3 Virology and Innate Immunity Research Group, Helmholtz Centre for Infection Research, Braunschweig, Germany; 4 Institute of Genetics, Technische Universität Braunschweig, Braunschweig, Germany; 5 Department of Biochemistry, University of Veterinary Medicine Hannover, Hannover, Germany; 6 Institute of Experimental Virology, TWINCORE, Centre for Experimental and Clinical Infection Research; a joint venture between the Hannover Medical School and the Helmholtz Centre for Infection Research, Hannover, Germany; 7 Department of Clinical Microbiology, Virology & Wallenberg Centre for Molecular Medicine (WCMM), Umeå University, Umeå, Sweden; 8 Leibniz Research Laboratories for Biotechnology and Artificial Organs (LEBAO), Department of Cardiothoracic, Transplantation and Vascular Surgery, REBIRTH—Center for Translational Regenerative Medicine, Biomedical Research in Endstage and Obstructive Lung Disease Hannover (BREATH), German Center for Lung Research (DZL), Hannover Medical School, Hannover, Germany; 9 Centre for Pathogen Evolution, Department of Zoology, University of Cambridge, Cambridge, United Kingdom; 10 Institute of Toxicology, Hannover Medical School, Core Facility Proteomics, Hannover, Germany; 11 Institute of Virology and Immunology (IVI), University of Bern, Bern, Switzerland; 12 Department of Infectious Diseases and Pathobiology, Vetsuisse Faculty, University of Bern, Bern, Switzerland; 13 Department of Tropical Disease Biology, Liverpool School of Tropical Medicine, Liverpool United Kingdom; Icahn School of Medicine at Mount Sinai, UNITED STATES

## Abstract

Inhibitors of bromodomain and extra-terminal proteins (iBETs), including JQ-1, have been suggested as potential prophylactics against SARS-CoV-2 infection. However, molecular mechanisms underlying JQ-1-mediated antiviral activity and its susceptibility to viral subversion remain incompletely understood. Pretreatment of cells with iBETs inhibited infection by SARS-CoV-2 variants and SARS-CoV, but not MERS-CoV. The antiviral activity manifested itself by reduced reporter expression of recombinant viruses, and reduced viral RNA quantities and infectious titers in the culture supernatant. While we confirmed JQ-1-mediated downregulation of expression of angiotensin-converting enzyme 2 (ACE2) and interferon-stimulated genes (ISGs), multi-omics analysis addressing the chromatin accessibility, transcriptome and proteome uncovered induction of an antiviral nuclear factor erythroid 2-related factor 2 (NRF-2)-mediated cytoprotective response as an additional mechanism through which JQ-1 inhibits SARS-CoV-2 replication. Pharmacological inhibition of NRF-2, and knockdown of NRF-2 and its target genes reduced JQ-1-mediated inhibition of SARS-CoV-2 replication. Serial passaging of SARS-CoV-2 in the presence of JQ-1 resulted in predominance of ORF6-deficient variant, which exhibited resistance to JQ-1 and increased sensitivity to exogenously administered type I interferon (IFN-I), suggesting a minimised need for SARS-CoV-2 ORF6-mediated repression of IFN signalling in the presence of JQ-1. Importantly, JQ-1 exhibited a transient antiviral activity when administered prophylactically in human airway bronchial epithelial cells (hBAECs), which was gradually subverted by SARS-CoV-2, and no antiviral activity when administered therapeutically following an established infection. We propose that JQ-1 exerts pleiotropic effects that collectively induce an antiviral state in the host, which is ultimately nullified by SARS-CoV-2 infection, raising questions about the clinical suitability of the iBETs in the context of COVID-19.

## Author summary

The COVID-19 pandemic has sparked interest in the therapeutic potential of iBETs, which was already being pursued to inhibit proliferation of cancer cells and reactivate HIV-1 latency. Prior studies reported that prophylactic administration of iBETs inhibits SARS-CoV-2 replication by downregulating ACE2 expression. However, a subsequent study reported that therapeutic administration of iBETs enhanced SARS-CoV-2 replication by inhibiting induction of the IFN signalling pathway. Despite this controversy, susceptibility of iBET-mediated anti-SARS-CoV-2 activity to viral subversion remains unknown. Here, we found that despite inhibiting the IFN signalling pathway, prophylactic administration of JQ-1 triggers a multi-pronged but transient anti-SARS-CoV-2 activity by downregulating ACE2 expression and inducing NRF-2-mediated cytoprotective response. This antiviral activity is limited to the *Sarbecoviruses*, as MERS-CoV was resistant. In contrast, therapeutic administration of JQ-1 did not inhibit SARS-CoV-2 replication. An ORF6-deficient variant—which exhibited resistance to JQ-1 and enhanced sensitivity to IFN-I treatment—emerged following serial passaging of SARS-CoV-2 under increasing concentrations of JQ-1. This work illuminates the imperfections of iBETs as potential candidates against COVID-19, but lays the foundation for their exploitation to steer SARS-CoV-2 virions towards IFN evolutionary traps and facilitate immune clearance.

## Introduction

There is an unmet need for novel effective therapies against the evolving variants of severe acute respiratory syndrome coronavirus-2 (SARS-CoV-2), the causative agent of the coronavirus disease-2019 (COVID-19) pandemic. The hallmark of severe SARS-CoV-2 infections is an excessive inflammatory response that results in tissue damage and multiorgan failure [1]. Therefore, therapeutic avenues that simultaneously dampen SARS-CoV-2 replication and antagonise its pathophysiological effects are utmostly desired.

A group of small molecular inhibitors of bromodomain and extra-terminal (BET) proteins (iBETs) has been suggested to hold potential for the realisation of such therapies [2–5]. The BET protein family is made up of four multifaceted, ubiquitously expressed and evolutionarily conserved proteins: bromodomain-containing protein (BRD) BRD2, BRD3, BRD4, and BRDT [6]. They act as epigenetic readers and transcriptional co-activators by binding to the acetylated lysine residues in histone tails in the chromatin and recruit the cellular transcriptional machinery to drive transcription of their target genes [7,8]. Alternatively, BET proteins also act as transcriptional co-repressors by interacting with their cellular partners to form repressor complexes, which suppress inappropriate transcriptional programs to maintain tissue homeostasis [8].

The ability of BET proteins to regulate transcription has made them prime targets for several diseases that hijack the cellular transcriptional machinery, including SARS-CoV-2 infection [9–13]. IBETs evict BET proteins from histones and their non-histone binding partners [14]. Therefore, clinical development of iBETs, most of which are in different phases of clinical trials in the context of cancer therapy and other pathologies [9,15], may provide a pharmacological tool for therapeutic intervention against several diseases that are driven by the BET protein-mediated transcriptional programs.

BRD2 co-activates expression of ACE2 [4], which is the main receptor for SARS-CoV-2 entry into target cells [16]. Consequently, treatment of target cells with iBETs downregulates ACE2 expression and inhibits SARS-CoV-2 replication and inflammatory responses [2–4]. Additionally, BET proteins co-activate the IFN [17] and nuclear factor kappa B (NF-κB) [18] signalling pathways, which are the main drivers of the inflammatory responses in COVID-19 patients [19,20].

Despite the growing literature on the anti-SARS-CoV-2 activity of iBET candidates [2–5], key questions remain regarding the genome-wide epigenetic alterations orchestrating iBET-mediated transcriptional responses that underlie their anti-SARS-CoV-2 activity. The missing information about the susceptibility of iBET-mediated antiviral activity to SARS-CoV-2 subversion is hampering their potential as the next generation of prophylactics in the context of COVID-19. Here, we sought to address these key questions by conducting an in-depth functional and multi-omics analysis of the signalling alterations that underlie JQ-1-mediated anti-SARS-CoV-2 activity.

## Materials & methods

### Ethics statement

Human bronchial airway epithelial cells (hBAECs) were isolated from explanted lungs obtained from the Hannover Lung Transplant Program after patient’s informed consent, ethical vote 2923–2015 from Hannover Medical School Ethical Committee, written consent was obtained from the patients.

### Chemicals and inhibitors

(+)-JQ-1 (Cat no. SML1524-5MG) was purchased from Sigma. ABBV-075 (Cat no. S8400) (ClinicalTrails.gov Identifier: NCT02391480), OTX015 (Cat no. S7360) (ClinicalTrails.gov Identifier: NCT02698176), ARV-825 (Cat no. S8297) and ML385 (Cat no. S8790) were purchased from Selleckchem. Dimethyl sulfoxide (DMSO) (Item no. 10127403) was purchased from Thermo Fisher Scientific. 4-octyl itaconate (4-OI) was purchased from MedChemExpress (Cat. no. HY-112675). Dimethyl fumarate (DMF) was purchased from Sigma (Cat. No. 227056). Ruxolitinib was purchased from InvivoGen (Cat. No. tlrl-rux). IFN-α2a (Roferon) was obtained from Roche.

### Cell lines

Calu-3 (ATCC HTB-55), Vero E6 (ATCC CRL-1586) and parental HEK293T cells (ATCC CRL-3216) were cultured in Dulbecco’s Modified Eagle’s Medium (DMEM) supplemented with 10% heat-inactivated fetal calf serum (FCS), 100 U/ml Penicillin-Streptomycin and 2mM L-glutamine (Gibco) (hereafter referred to as 10% DMEM) at 37°C/5% CO_**2**_. Unless stated otherwise, all cell cultures were maintained at 37°C/5% CO_**2**_.

### Human bronchial airway epithelial cells (Air-liquid interface cultures)

Human bronchial airway epithelial cells (hBAECs) were isolated from explanted lungs obtained from the Hannover Lung Transplant Program after patient’s informed consent, ethical vote 2923–2015 from Hannover Medical School Ethical Committee, written consent was obtained from the patients. For isolation of hBAECs, human bronchial tissue was cut into small pieces in Hank’s buffer (Thermo Fisher Scientific) containing 0.18% protease XIV and incubated for two hours at 37°C. After thorough pipetting with a 25/50 ml serological pipette, cell solution was filtered through a 100 μm cell strainer (Corning) to remove clumps and 10 ml RPMI supplemented with 10% FCS (Thermo Fisher Scientific) was added. After centrifugation for 10 min at 500*g* and 4°C, supernatant was removed and cells were resuspended in SAEGM (PromoCell) + Primocin (InvivoGen) + Penicillin-Streptomycin (P/S) (Sigma-Aldrich). For ALI cultures, 200,000 hBAECs were seeded onto PureCol- (Advanced BioMatrix) coated 12-well inserts (Greiner Bio-One) in SAEGM + Primocin + P/S. 48 hours post seeding, culture medium in apical and basal chambers was changed to PneumaCult-ALI medium (STEMCELL Technologies). Air lift was performed 48 hours later by gently removing medium from the apical chamber. Homogenous distributed cilia were visible three weeks after air lift and inserts were used for infection experiments.

### Cell viability assays

Calu-3 cells (6x10^5^ cells/ml) seeded overnight in 96-well plates were treated with serial dilutions (prepared in culture medium) of the iBETs or corresponding DMSO controls for 72 hours; with PBS wash, medium change and fresh drug administration every 24 hours. Post-treatment, cells were subjected to viability assays using the CellTiter-Glo Luminescent Cell Viability Assay Kit (Promega) for Calu-3 cells and CellTiter-Glo 3-D Cell Viability Assay Kit (Promega) for hBAECs according to the manufacturer’s protocol. The raw data from the test samples were background-subtracted, normalised to naïve cells, and analysed using GraphPad Prism v9 (LaJolla, CA, USA) as previously described [21].

### Virus production

SARS-CoV-2 [passage (P) 1, BetaCoV/Munich/BavPat1/2020|EPI_ISL_406862] [22], SARS-CoV (P1, HKU-39849 Hong Kong) [23,24], and MERS-CoV (P2, EMC/2012) [25] stocks from isolates were propagated in Vero E6 cells to generate P2 and P3 stocks, respectively [26]. Recombinant SARS-CoV-2 rB.1 (NC_045512.2 with spike mutation D614G) [27] as well as a rB.1 ORF6:stop2 (isogenic virus with a stop codon at position 2 of ORF6) [28], and GFP-tagged SARS-CoV-2 (synSARS-CoV-2-GFP-P2A-ORF7a clone #41) [29] were produced as previously described [27]. All infectious pathogens were handled under biosafety level three (BSL-3) conditions with respiratory personal protection equipment.

### Next generation sequencing of virus stocks

Viral RNA from the supernatant was extracted using the NucleoSpin RNA Virus Isolation Kit (Macherey-Nagel) according to the manufacturer’s protocol. The RNA-seq library was prepared from viral RNA extracts using the KAPA RNA HyperPrep Kit (Roche, Penzberg, Germany) and KAPA DI adaptors according to the manufacturers’ instructions. The RNA library was subjected to next generation sequencing (NGS) on a NextSeq System (Illumina) using a NextSeq 500/550 v2.5 Kit (Illumina). Sequences were analysed using the Geneious v9.1.8, and assembled by mapping reads to the reference sequence. We only used stocks with intact furin cleavage site and virtually absent (<5%) minority variants.

To determine the minor variant frequency of TAA stop codon mutations in ORF6, sequencing reads were aligned against the SARS-CoV-2 Wuhan reference sequence (GenBank accession NC_045512.2) using Bowtie2 (version 2.4.2) [30] by applying its local alignment option and default parameters. From the resulting SAM files, samtools (version 1.17) [31] was used to extract reads overlapping the genome nucleotide locations of interest (27217–27219). Reads with a nucleotide PHRED quality score of less than 30 were ignored. Reads with soft-clipped nucleotides overlapping the region of interest were retained, unless all three locations were soft-clipped. This retains reads that carry a mutation in the target region, which should be included in the stop codon counting, but were marked as soft-clipped by Bowtie2 due to mismatch with the reference sequence at one or two locations at the end of the read. Ignoring reads where all three target locations were soft-clipped prevents counting reads with a significant mismatching soft-clipped region, which may be far from the matched part of the read. Codon frequencies were then calculated from all acceptable reads.

### Virus infection of Calu-3 cells

Calu-3 cells (6x10^5^ cells/ml) seeded overnight in 12-well plates were treated with serial dilutions of iBETs or corresponding DMSO controls for 48 hours; with PBS wash, medium change and fresh drug administration every 24 hours. Post-treatment, cells were inoculated with viruses (SARS-CoV and SARS-CoV-2, MOI 0.1; and MERS-CoV, MOI 0.0001) and incubated for one hour. After infection, cells were washed with PBS, supplied with fresh medium and iBETs, and incubated for 24 hours. The next day, 50 μl of supernatant was collected into 300 μl of RAV1 lysis buffer (Machery-Nagel) for viral RNA extraction and 100 μl into 100 μl of 0.5% gelatin medium for plaque assays. Cells were washed with PBS, trypsinized and reconstituted in culture medium, from which 50 μl was collected into 300 μl of RAV1 lysis buffer (Machery-Nagel) to isolate cell-associated RNA. RNA samples were stored at -20°C and plaque assay samples at -80°C until processing.

### Virus infection of hBAECs

JQ-1 or DMSO was added into the culture medium (PneumaCult-ALI) in the basal compartment of cells cultured in transwells and incubated for 48 hours, followed by medium exchange and new drug administration after 24 hours as mentioned above. Post-treatment, cells were washed three times with pre-warmed PBS to remove mucus from the apical compartment and inoculated with SARS-CoV-2 (2x10^4^ PFU diluted in OptiPRO medium), followed by incubation for 90 minutes. The inoculum was then aspirated and cells washed with PBS three times. The transwells were then transferred into new plates with the drug-containing culture medium. To harvest samples for zero time point, 200 μl of culture medium was immediately added onto the cells in the apical compartment and incubated for 15 min to elute the virus particles. Following incubation, 50 μl of virus eluate was collected into 300 μl RAV1 lysis buffer (Machery-Nagel) for viral RNA extraction and 100 μl into 100 μl of 0.5% gelatin medium for plaque assays, after which the eluate residues were aspirated from the apical compartment and followed by incubation of cells. This harvesting procedure was repeated every 24 hours for kinetic experiments. The samples were stored at -20°C and -80°C, respectively, until processing.

### Quantitative real-time PCR (qRT-PCR)

Viral RNA from the supernatant was extracted using the NucleoSpin RNA Virus Isolation Kit (Macherey-Nagel) according to the manufacturer’s protocol. To quantify viral RNA copies, a 12.5 μl reaction/well was prepared in 4titude 96-Well Semi-Skirted PCR Plates for LC480 (Roche) using a SuperScript III One-Step qRT-PCR Kit (Invitrogen). Reverse transcription of the viral RNA and amplification of the cDNA was conducted in a LightCycler 480 System (Roche) using the following protocol: 55°C, 10 min; 95°C, 3 min; 95°C, 15 sec; 58°C, 30 sec and 40°C, 30 sec for 45 cycles. Primers and probes designed against SARS-CoV and SARS-CoV-2 envelope (E) gene [32], and the region upstream of MERS-CoV E gene (upE) [33] were used. The absolute quantification of the viral RNA copies was calculated by a standard curve method using viral RNA standards [32,33]. Unless stated otherwise, the qRT-PCR data from coronavirus-infected cells are expressed as E Copies/μl RNA. To analyse human ISG expression, RNA was extracted using the Direct-Zol RNA MiniPrep Kit (Zymo Research). The extracted RNA was subjected to cDNA synthesis (NEB, Invitrogen). Quantification of relative mRNA levels was performed with the LightCycler 480 Instrument II (Roche) using Taq-Man PCR technology. For human IFN-induced protein with tetratricopeptide repeats 1 (IFIT1) and MX dynamin like GTPase 2 (MX2), premade primer-probe kits were used (Applied Biosystems, assay IDs: Hs01911452_s1; Hs01550814_m1, respectively). Relative mRNA levels were determined by the ΔΔCt method using human RNASEP (Applied Biosystems) as the internal reference. Each sample was analysed in technical triplicates and with parallel controls without the reverse transcriptase. Data analysis was performed using LightCycler Software 4.1 (Roche).

### Plaque assays

Vero E6 cells (4 x 10^5^ cell/ml) were seeded overnight in 24-well plates. The virus-containing samples (50 μl) were serially diluted in 450 μl aliquots of serum-free Opti-Pro medium (Gibco), after which 200 μl from each dilution were added in duplicates to the cells, followed by one-hour incubation. Post-infection, viral inoculum was aspirated from the cells, cells washed with PBS, and overlaid with 2.4% Avicel (FMC BioPolymers) mixed with 2xDMEM (1:1 ratio), followed by incubation for 72 hours. The overlay was aspirated from the cells, after which they were fixed for 30 min with 6% formaldehyde and stained with crystal violet solution (0.2% crystal violet, 2% ethanol and 10% formaldehyde) for 20 min. Plaque Forming Units (PFU) were determined from at least two dilutions for which distinct plaques were detectable. The data from the plaque assays are expressed as PFU/ml.

### Flow cytometry

JQ-1 and DMSO-treated Calu-3 cells were infected with GFP-expressing SARS-CoV-2 (synSARS-CoV-2-GFP-P2A-ORF7a clone #41) [29] (MOI 0.25) following the above-mentioned protocol. Subsequently, cells were trypsinized, PBS-washed and fixed for 90 minutes with 4% PFA. GFP signal was quantified by flow cytometry in FACSCelesta (BD Bioscience) and analysed by FlowJo v10.8 (Tree Star, Ashland, Oregon, USA).

### Immunoblotting

Calu-3 cells were lysed in 60 μl RIPA Lysis Buffer (Thermo Fisher Scientific) supplied with 1% Protease Inhibitor Cocktail Set III (Merck Chemicals) for 30 min at 4°C. Subsequently, cell lysates were pelleted and protein concentration in supernatant was determined by BCA protein assay (ThermoFisher Scientific). 20 μg total protein from each sample was mixed with 4x Laemmli buffer, which was supplemented with 10% beta-mercaptoethanol, then boiled for ten minutes at 95°C to ensure protein denaturation. Proteins were resolved on 10% SDS-PAGE gel and transferred to a nitrocellulose membrane (0.45 μm pore size, GE Healthcare) by Trans-Blot Turbo System (BioRad). Membranes were blocked with 5% dried milk in 0.1% PBS-Tween (0.9% NaCl, 10 mM Tris-HCl [pH 7.5], 0.1% Tween 20) for 30 min at room temperature. Blocked membranes were incubated with the primary antibodies against ACE2 (#AF933, R&D Systems), SARS-CoV ORF6 (Enjuanes’s Laboratory, Spain), SARS-CoV-2 nucleocapsid (#GTX 135361, Stratech), NRF-2 (#12721, Cell Signalling Technology), HO-1 (#5853, Cell Signalling Technology) NQO-1 (#3187, Cell Signalling Technology), KEAP-1 (#8047, Cell Signalling Technology), ISG15 (#sc-166755, SantaCruz), IFIT1 (#TA500948S, OriGene), tubulin (#2144S, Cell Signalling Technology) and beta-actin (#A5316, Sigma-Aldrich). Secondary antibodies conjugated with horseradish peroxidase (HRP) were used for chemiluminescence-based detection by Fusion Fx7 (Peqlab Biotechnologie GmbH). Detection was performed using SuperSignal West Femto substrate (ThermoFisher Scientific).

### siRNA-mediated knockdown in Calu-3 cells

Calu-3 cells were seeded at a density of 2.5x10^5^ cells/ml in 12-well plates. 24 and 72 hours post seeding, cells were transfected twice with 10 μM of either non-targeting small-interfering RNA (siRNA) (sc-37007) or NRF-2- (sc-37030), NQO-1- (sc-37139) or HO-1- (sc-35554) specific siRNA using Lipofectamine RNAiMAX (Thermo Fisher) according to the manufacturer’s instructions. 72 and 96 hours after seeding, cells were pretreated with 0.256% DMSO and 2.56 μM JQ-1. 48 hours after the second transfection, Calu-3 cells were infected with SARS-CoV-2 (MOI of 0.1). One hour post-infection, viral inoculum was removed, cells were washed with PBS and supplemented with fresh media. 24 hours post-infection, supernatants and cells were harvested for qRT-PCR, plaque assay and immunoblot analysis, respectively. NRF-2, NQO-1 and HO-1 protein expression levels were measured by immunoblotting.

### Production of pseudotyped lentivirus particles and transduction assays

SARS-CoV-2 spike (S)- and VSV-G-pseudotyped lentiviral particles [34] were produced by calcium phosphate-based transfection of HEK293T cells with the packaging plasmid pCMV ΔR8.91 [35], the lentiviral transfer plasmid pCSII-EF-luciferase [36], and pCMV-VSV-G [37] using the CalPhos Mammalian Transfection Kit (Takara Bio Company) according to the manufacturer’s protocol. Dose-dependent treatment of Calu-3 cells with JQ-1, followed by transduction with pseudovirus particles in 96-well plates was conducted under BSL-2 conditions, after which the infection efficiency was analysed luminometrically in Synergy HTX Multi-Mode Microplate Reader (BioTek Instruments, Inc.) using the Luciferase Assay System (Promega) according to the manufacturer’s protocol.

### ATAC-seq

Bulk ATAC-seq and RNA-seq were conducted in parallel from the same infection experiment. Calu-3 cells were treated with JQ-1 and DMSO, respectively, for 24 hours, infected with SARS-CoV-2 (MOI 0.1) under continuous treatment for another 24 hours. Uninfected but treated, and naïve Calu-3 cells were used as controls. Post-infection, ATAC-seq libraries were prepared from 50,000 cells per replicate using Illumina Tagment DNA Enzyme and Buffer Kit (#20034197 Illumina) according to the Omi-ATAC-seq protocol [38] with minor optimizations. Briefly, 5 μl from the partially amplified barcoded fragments was subjected to SYBR Green qRT-PCR in a LightCycler 480 System (Roche) using the FastStart Essential DNA Green Master Mix (Roche) according to the manufacturer’s protocol. Amplification was conducted for 20 cycles using a universal forward primer and the sample-specific barcoded reverse primers [39]. Amplification curves from SYBR Green qRT-PCR were generated and used to determine the number of cycles that give ⅓ of the maximum fluorescence. Final library preparation was conducted by conventional PCR for 8–12 cycles. The amplified libraries were subjected to a single left-sided bead purification using the AMPure XP magnetic beads (Beckman Coulter). Libraries were sequenced on SP lane of NovaSeq 6000 System (Illumina) at the MDC/BIH Genomic Core Facility to generate 40 million 75-nucleotide paired-end reads per sample.

### RNA-seq

Total RNA was isolated from 3x10^5^ cells per replicate using Direct-Zol RNA Miniprep Kit (Zymo Research) according to the manufacturer’s protocol and shipped in dry ice to the MDC/BIH Genomic Core Facility for quality assessment using TapeStation (Agilent Technologies). Libraries were prepared using TrueSeq Stranded mRNA Kit to generate Illumina-compatible libraries by following the manufacturer’s protocol (Illumina). Libraries were sequenced on SP lane of NovaSeq 6000 System (Illumina) to generate 40 million 75-nucleotide paired-end reads per sample.

### Liquid chromatography tandem mass spectrometry (LC-MS/MS)

Treatment of Calu-3 cells and infection with SARS-CoV-2 were performed following the above-mentioned protocol. Treated but uninfected and naïve cells served as controls. Preparation of cell lysate samples and protein quantification were conducted as mentioned above, from which 50 μg of proteins per sample were prepared and resolved on 7.5% linear SDS-PAGE gels as described elsewhere [21]. The in-gel protein digestion and liquid chromatography coupled with tandem mass spectrometry (LC-MS/MS) experiments were performed as previously described [40].

### Bioinformatics analysis

For RNA-seq, reads were aligned to the human genome version GRCh38 and counted using STAR [41]. Differential expression analysis was conducted using DESeq2 version 1.30 [42]. Transcription factor analysis was performed using the R package Dorothea [43]. For ATAC-seq, reads were aligned to the human genome GRCh38, after which the peaks were called using MACS2 v. 2.2.7.1 [44]. For differential TF binding analysis, the R package DiffBind v. 3.0.15 [45] was used. Motif search analysis was performed using the MEME Suite [46] and DREME algorithm (https://meme-suite.org/meme/doc/dreme.html). Gene set enrichment analysis was performed with the R package cluster Profiler v. 3.18 [47]. All results were corrected for multiple testing using the Benjamini-Hochberg procedure [48]. Genes were annotated as involved in IFN signalling, the cell cycle, or autophagy by referring to the Reactome Interferon Signalling geneset (R-HSA-913531), the Reactome Cell Cycle geneset (R-HSA-1640170), and the KEGG Autophagy—animal geneset (hsa04140), respectively. Genes identified as targets for NRF-2 signalling were identified based on a previous report [49]. LC-MS/MS data analysis was conducted as previously described [21].

### Statistical analysis

Unless stated otherwise, data are presented as the mean ± standard error of the mean (SEM) from the indicated number of independent experiments. Statistical significance was calculated by performing unpaired parametric t-tests for comparisons between two groups and One-Way ANOVA for multi comparisons using GraphPad Prism v.10. *P* values ≤0.05 were considered significant: <0.0332 (*), <0.0021 (**), <0.0002 (***), <0.0001 (****) and ns = not significant (>0.05).

## Results

### Prophylactic administration of iBETs inhibits SARS-CoV-2 infection

**Fig 1:**
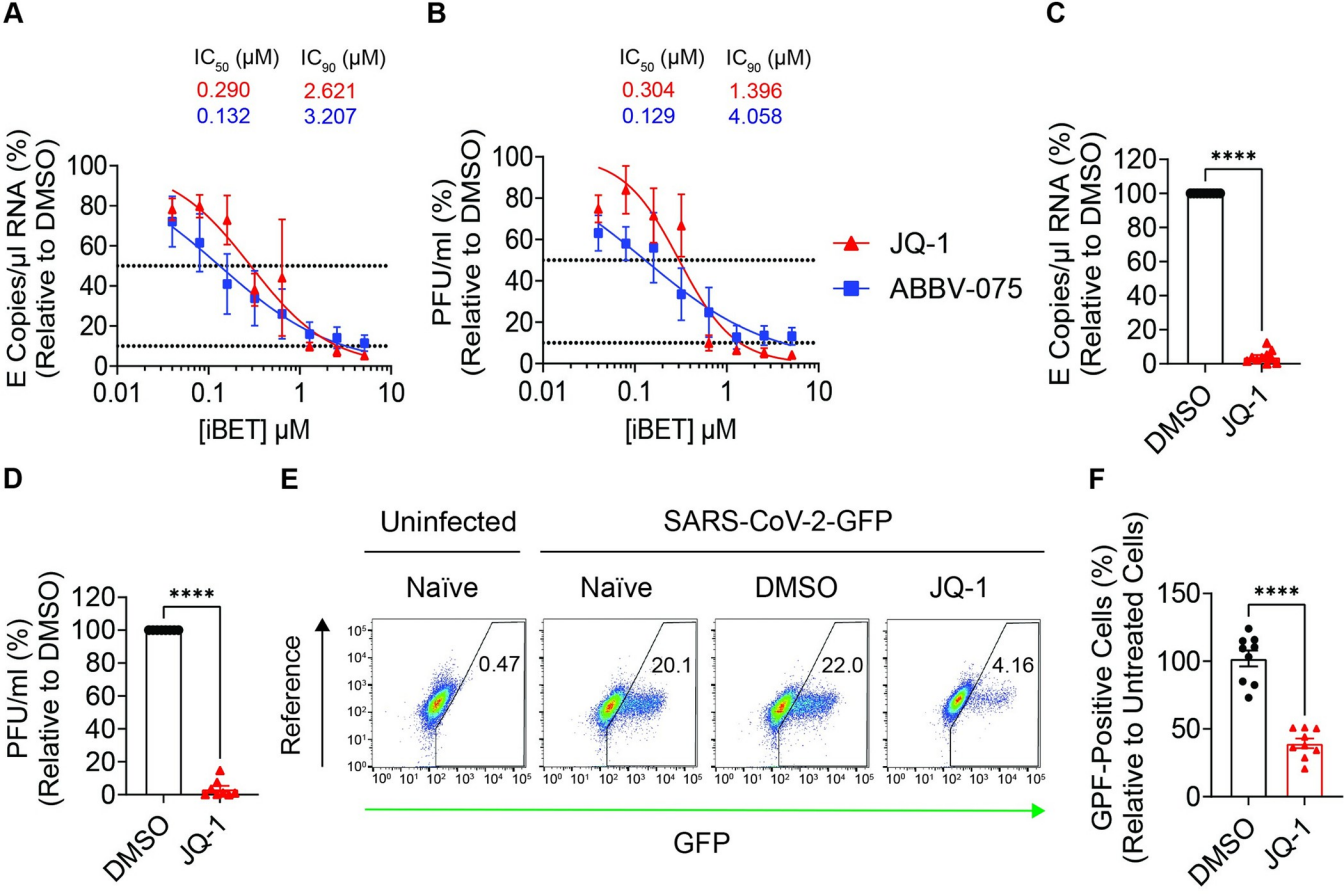
Prophylactic administration of iBETs inhibits SARS-CoV-2 infection. (**A-B**) Dose response curves (n = 3) showing the effect of the indicated iBETs on the quantities of (**A**) SARS-CoV-2 genomic RNA (E copies/μl) and (**B**) infectious titers (PFU/ml) in the supernatant at 24 h.p.i. Calu-3 cells were pretreated for 48 hours prior to infection with SARS-CoV-2 (MOI = 0.1) for 24 hours under continuous presence of the drug. (**C-D**) Relative quantification of SARS-CoV-2 (**C**) genomic RNA (E copies/μl) and (**D**) titers (PFU/ml) in the supernatants harvested from the apical compartment of hBAECs at 48 h.p.i. Cultures were pretreated with DMSO or JQ-1 (2.56 μM) for 48 hours and infected with SARS-CoV-2 (2x10^4^ PFUs) for 48 hours under continuous presence of the drug. Unpaired parametric t-test was used to compare the means from duplicates of five and four independent experiments, respectively. (**E**) Representative FACS dot plots and (**F**) relative quantification of GFP-positive cells in cell populations that were treated with DMSO or JQ-1 (2.56 μM) prior to infection with SARS-CoV-2-GFP (MOI = 0.25). Calu-3 cells were pretreated for 48 hours prior to infection with SARS-CoV-2 (MOI = 0.1) for 24 hours under continuous presence of the drug. GFP expression was quantified by flow cytometry and the data are shown as GFP-positive cells from treated cells relative to untreated cells. Unpaired parametric t-test was used to compare the means from triplicates of three independent experiments. Raw data are shown in **S1 Data**.

Inhibition of SARS-CoV-2 infection by several iBET compounds has been reported in different infection models [2–4]. To gain more insights into their antiviral potency in the context of SARS-CoV-2 infection, we compared antiviral activity of four iBETs: three (JQ-1, ABBV-075, and OTX-015) of which compete with the acetylated molecules for binding to the bromodomains of the BET proteins, and one degrader of BET proteins (dBET) (ARV-825) that carries proteolysis-targeting chimera (PROTAC) sequence to mark BET proteins for proteasomal degradation. IBET treatment of Calu-3 cells prior to SARS-CoV-2 infection led to a dose-dependent reduction of viral genomic RNA quantities (Figs 1A and S1C) and infectious titers (Figs 1B and S1D) in the supernatant in the absence of detectable cell toxicity (S1 A and S1E Fig). Of the investigated iBET candidates, JQ-1 (IC_50_ = 0.290 μM) and ABBV-075 (IC_50_ = 0.132 μM) were more potent than OTX-015 (IC_50_ = 3.553 μM) and ARV-825 (IC_50_ = 1.431 μM) against SARS-CoV-2 infection in Calu-3 cells. Prophylactic administration of JQ-1 to hBAECs reduced SARS-CoV-2 viral RNA quantities by 24.8-fold (Fig 1C) and infectious titers by 28.2-fold (Fig 1D) without detectable toxicity (S1B Fig), demonstrating the relevance of iBET-mediated anti-SARS-CoV-2 activity in a physiologically relevant primary cell model [4]. JQ-1 treatment rendered Calu-3 cells 2.6-fold less susceptible to infection by recombinant GFP-expressing SARS-CoV-2, reflecting the consistency of JQ-1-mediated anti-SARS-CoV-2 activity across different readouts (Fig 1E–1F).

### JQ-1 exhibits a subgenera-specific antiviral activity among betacoronaviruses

**Fig 2:**
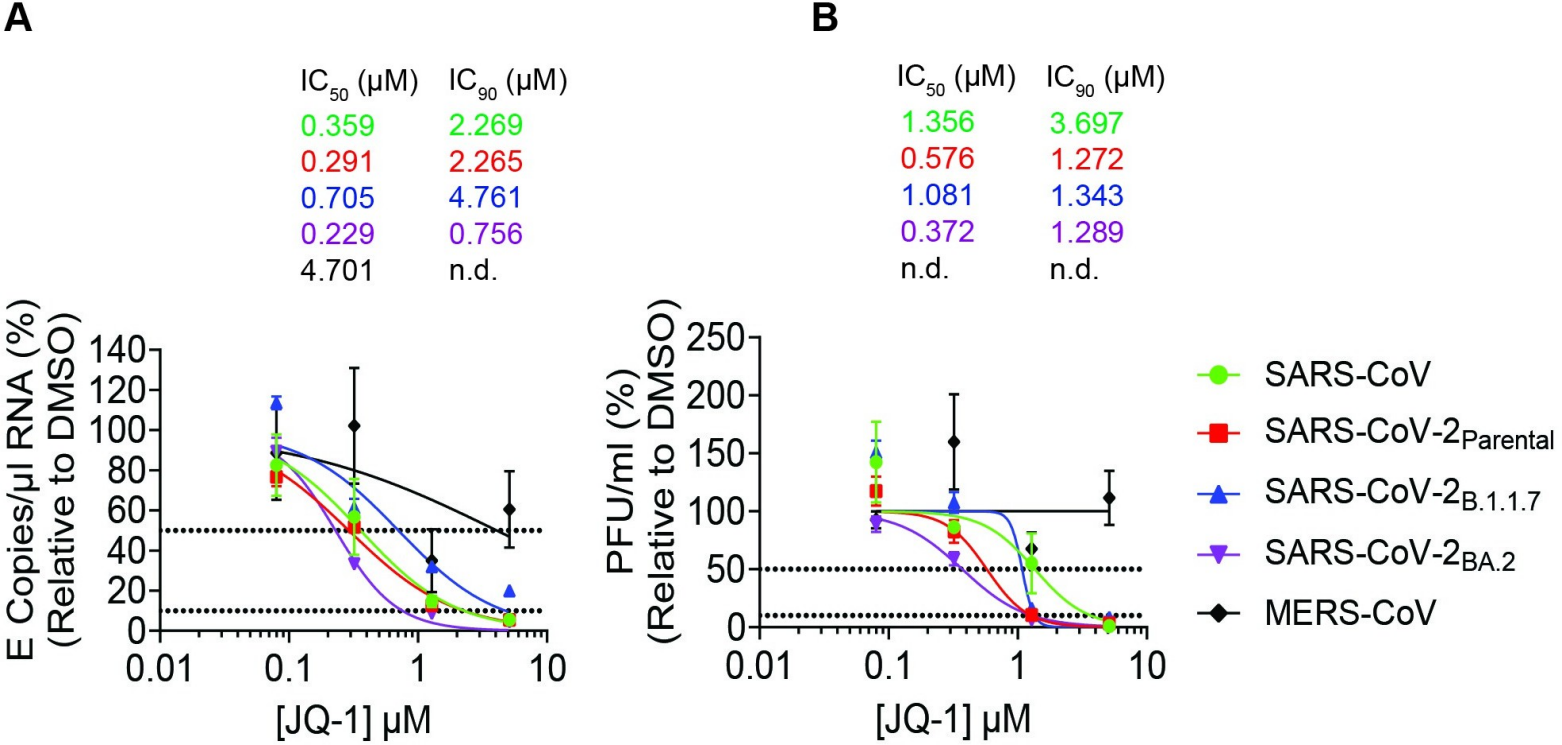
JQ-1 exhibits a subgenera-specific antiviral activity among betacoronaviruses. **(A-B)** Dose response curves (n = 3) showing the effect of JQ-1 against the indicated coronaviruses on the quantities of (**A**) coronaviral genomic RNA (E copies/μl) and (**B**) infectious titers (PFU/ml) in the supernatant at 24 h.p.i. Calu-3 cells were pretreated twice for 48 hours prior to infection with *Sarbecoviruses* (MOI = 0.1) and MERS-CoV (MOI = 0.0001) for 24 hours under continuous presence of the drug. Raw data are shown in **S1 Data**.

To investigate whether the iBET-mediated anti-SARS-CoV-2 activity extends beyond SARS-CoV-2 parental strains, which have been the main focus of the previous studies [2–4], we compared the antiviral potency of JQ-1 in Calu-3 cells infected with a panel of ß-coronaviruses. JQ-1 potently and dose-dependently inhibited infection by SARS-CoV, SARS-CoV-2_B.1_, SARS-CoV-2_B.1.1.7_ and SARS-CoV-2_BA.2_, in contrast to MERS-CoV, as indicated by the reduction of viral RNA copies (Fig 2A) and infectious titers (Fig 2B) in culture supernatants. These data suggest that JQ-1 exhibits a subgenera-specific antiviral activity, which is directed towards infection by *Sarbecoviruses* and not *Merbecoviruses* to which MERS-CoV belongs.

### JQ-1 exhibits a cell-directed anti-SARS-CoV-2 activity

**Fig 3:**
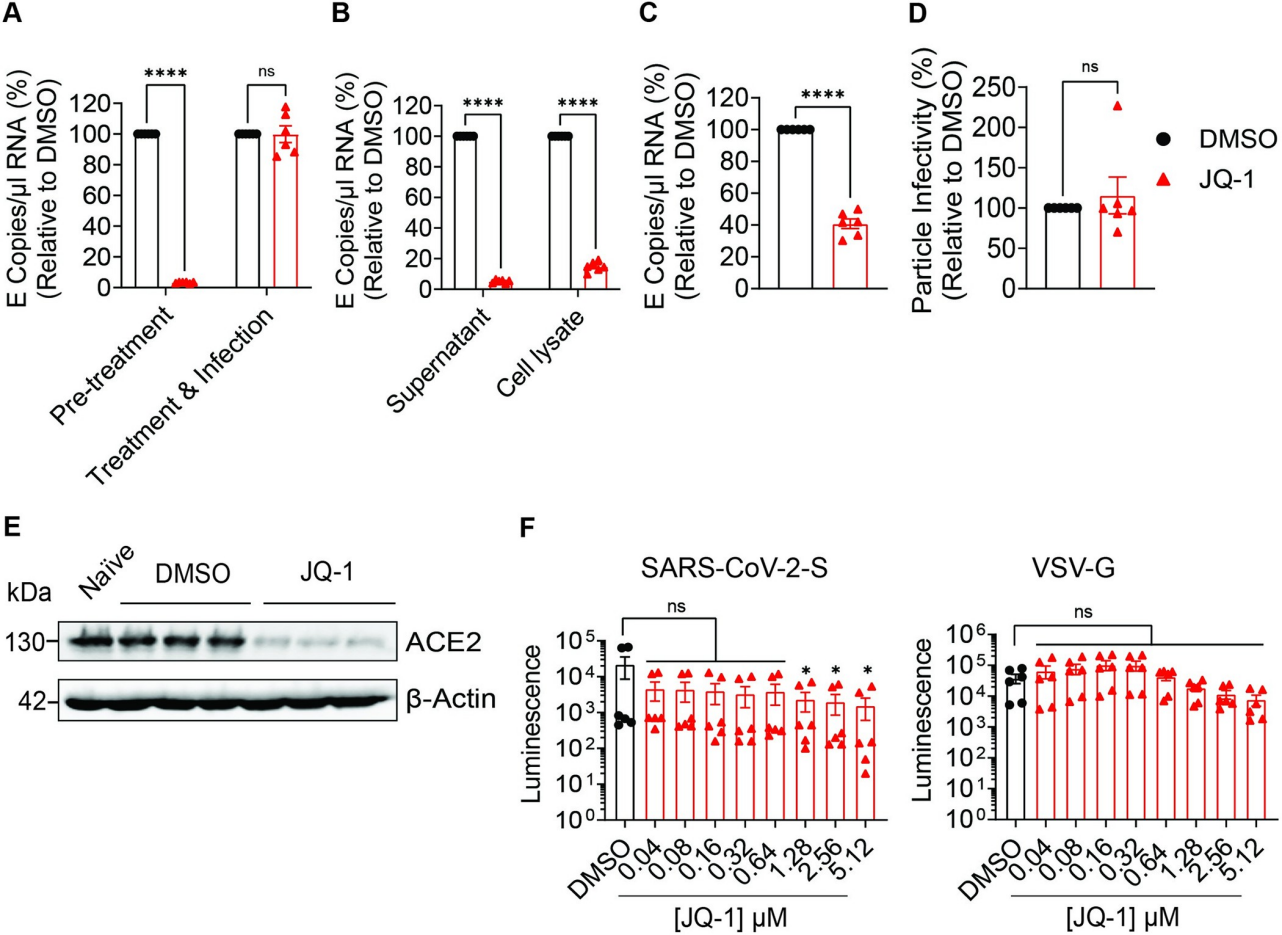
JQ-1 exhibits a cell-directed anti-SARS-CoV-2 activity. (**A**) Relative quantification of SARS-CoV-2 genomic RNA (E copies/μl) in the supernatants of Calu-3 cells that were treated prior to infection (Pre-treatment) or at the time of infection (Treatment & Infection). Calu-3 cells were pretreated twice for 48 hours with DMSO or JQ-1 (2.56 μM) prior to infection or treated at the time of infection with SARS-CoV-2 (MOI = 0.1) for 24 hours under continuous presence of the drug. (**B**) Relative quantification of SARS-CoV-2 genomic RNA (E copies/μl) in the supernatants and cell lysates of Calu-3 cells. Calu-3 cells were pretreated twice for 48 hours with DMSO or JQ-1 (2.56μM) prior to infection with SARS-CoV-2 (MOI = 0.1) for 24 hours under continuous presence of the drug. Unpaired parametric t-test with the Holm-Šídák correction for multiple testing was used to compare the means from duplicates of three independent experiments. Relative quantification of SARS-CoV-2 particle (**C**) release and (**D**) infectivity in Calu-3 cells treated with with DMSO or JQ-1 (2.56μM) prior to infection (MOI = 0.1) under continuous presence of the drug. Viral particle release was calculated using this formula: Release = Viral RNA_supernatant_/Viral RNA_supernatant_ + Viral RNA_cell lysate_. Particle infectivity was calculated using this formula: Infectivity = Viral Titers_supernatant_/Viral RNA_supernatant_. Unpaired parametric t-test was used to compare the means from duplicates of three independent experiments. (**E**) Immunoblot analysis of ACE2 expression in Calu-3 cells treated twice with DMSO or JQ-1 (2.56μM) for 48 hours. (**F**) Luminometric quantification of transduction efficiency of DMSO or JQ-1-treated Calu-3 cells using luciferase-expressing lentiviral pseudoparticles decorated with SARS-CoV-2-S and VSV-G proteins. Calu-3 cells were pretreated twice for 48 hours with DMSO and indicated concentrations of JQ-1 prior to transduction for 24 hours under continuous presence of the drug. One-way ANOVA with Dunnett’s multiple comparison test was used to compare the means from duplicates of three independent experiments. Raw data are shown in **S1 Data**.

Next, we compared the effect of JQ-1-mediated anti-SARS-CoV-2 activity when administered prophylactically and at the time point of infection. JQ-1 inhibited SARS-CoV-2 replication when administered prophylactically in Calu-3 cells, while it failed to inhibit infection when administered to cells at the time of infection (Fig 3A). qRT-PCR-based quantification of viral RNA copies showed that JQ-1-mediated reduction of viral RNA copies in the supernatant of infected Calu-3 cells is accompanied by, and probably a consequence of, reduced quantities of cell-associated viral RNA (Fig 3B). The ratio of extracellular to total viral RNA indicated that JQ-1 impaired release of SARS-CoV-2 virions (Fig 3C), without *per se* reducing the specific particle infectivity of secreted viral particles (Fig 3D). In accordance with previous reports [2–4], JQ-1 treatment reduced steady-state levels of ACE2 expression (Fig 3E), an effect that was accompanied by reduced susceptibility of Calu-3 cells to transduction with SARS-CoV-2 spike, but not VSV-G-pseudotyped lentiviral particles (Fig 3F). Despite the fact that lentiviral pseudotypes do not fully recapitulate the entry process of authentic virions, these data suggest that the cell-directed anti-SARS-CoV-2 activity of JQ-1 involves, but likely is not restricted to, downregulation of ACE2 expression.

### SARS-CoV-2 infection and JQ-1 treatment modulate the chromatin regulatory landscape

**Fig 4:**
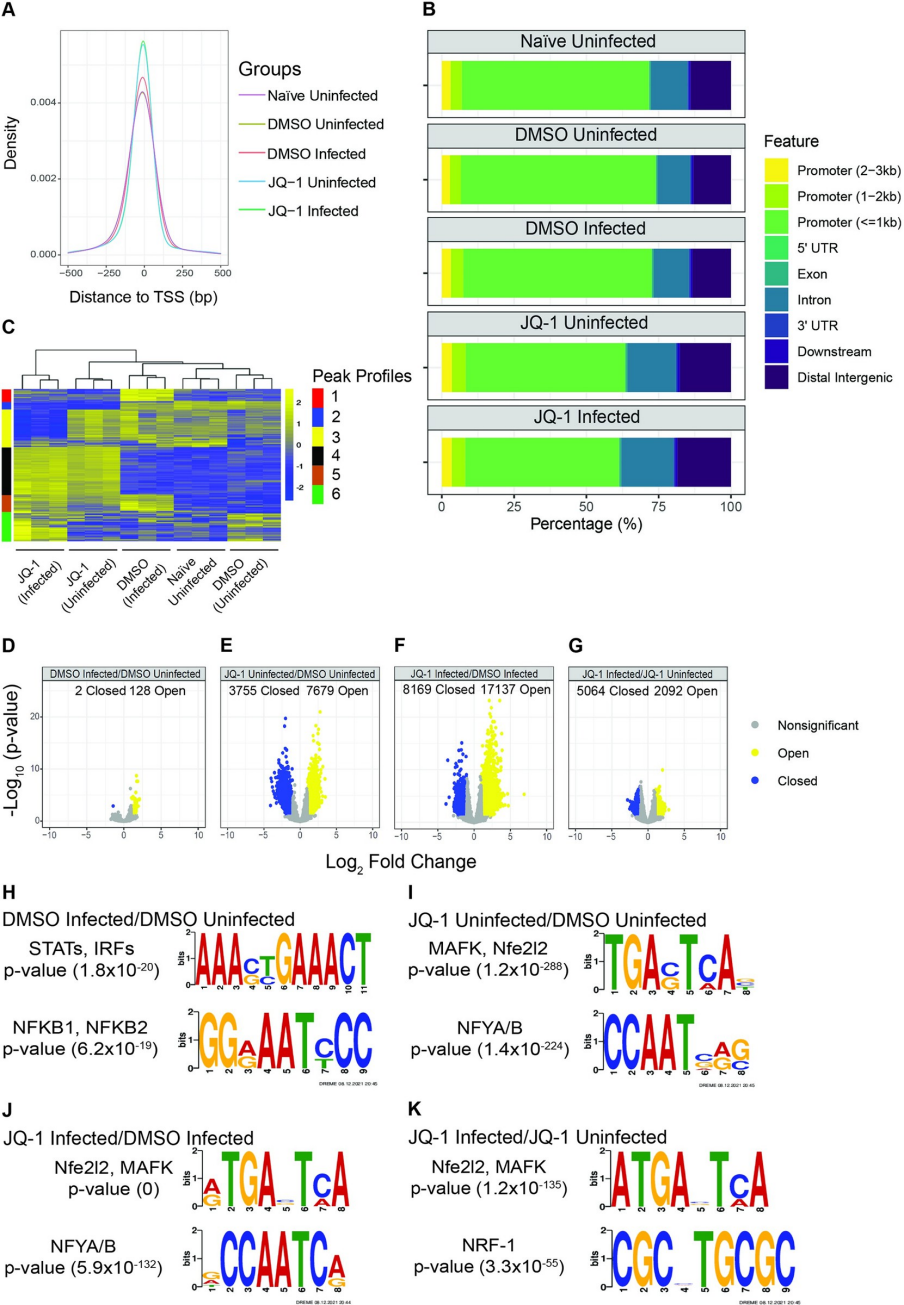
SARS-CoV-2 infection and JQ-1 treatment modulate the chromatin regulatory landscape. (**A**) TSS plot depicting the density and distribution of accessible ATAC-seq peaks around transcription start sites within a window of -500 to 500 bp. (**B**) Analysis of the genomic features annotated to accessible ATAC-seq peaks in the chromatin. (**C**) Heatmap of differentially accessible ATAC-seq peaks scaled as z-score across rows. (**D-G**) Volcano plots showing relative log_2_ fold change (log_2_FC) and statistical significance [-log_10_ (p-value)] of differentially accessible ATAC-seq peaks in (**D**) DMSO-treated infected/DMSO-treated uninfected, (**E**) JQ-1-treated uninfected/DMSO-treated uninfected, (**F**) JQ-1-treated infected/DMSO-treated infected, and (**G**) JQ-1-treated infected/JQ-1-treated uninfected contrasts. Significantly (FDR of ≤0.05) regulated peaks are indicated. (**H-K**) TF motif enrichment of accessible ATAC-seq peaks in (**H**) DMSO-treated infected/DMSO-treated uninfected, (**I**) JQ-1-treated uninfected/DMSO-treated uninfected, (**J**) JQ-1-treated infected/DMSO-treated infected and (**K**) JQ-1-treated infected/JQ-1-treated uninfected contrasts. The TF motifs were identified using the DREME algorithm, where the height of the letters represents the frequency of each base in the motif.

To investigate the chromatin regulatory landscape that orchestrates JQ-1-mediated SARS-CoV-2 inhibition, we subjected Calu-3 cells to bulk ATAC-seq. Principal component analysis (PCA) showed clustering of samples according to their experimental groups, reflecting comparable chromatin profiles between individual samples in each group (S2A Fig). JQ-1 treatment induced larger changes than SARS-CoV-2 infection. Samples from uninfected DMSO-treated cells closely clustered with samples from naïve cells, suggesting that DMSO treatment induced minimal changes to the host chromatin profile. Compared to the control groups, JQ-1 administration to Calu-3 cells increased accessibility to the transcriptional start sites (TSS) irrespective of the infection status, followed by SARS-CoV-2 infection, as indicated by the increase in the density of peaks mapping to the TSSs (Fig 4A), illustrating the superiority of JQ-1 treatment in remodelling chromatin accessibility to the TSSs compared to SARS-CoV-2 infection. Annotation of the accessible peaks to the genomic features in the chromatin showed a large coverage for promoter sequences located within 1 kb from the TSS regions across all experimental groups (Fig 4B). However, irrespective of the infection status, JQ-1 treatment shifted a proportion of the accessible regions away from the promoter sequences located within 1 kb from the TSS regions to the introns and distal intergenic regions. This suggests that regulation of transcription under JQ-1 treatment is driven by a more evenly-distributed accessibility of genomic features compared to SARS-CoV-2 infection or absence of JQ-1, where transcriptional regulation is predominantly driven by the proximal regulatory cis-elements in the promoters. Hierarchical clustering of significantly regulated peaks in each experimental group revealed distinct chromatin accessibility profiles, modulated distinctly by either SARS-CoV-2 infection or JQ-1 treatment, with some commonly upregulated peaks resulting from both SARS-CoV-2 infection and JQ-1 treatment, respectively (Fig 4C).

Volcano plots of the ATAC-seq data revealed that SARS-CoV-2 infection only mildly modulated the chromatin accessibility profile (significant changes in 130 ATAC-seq peaks, 128 of which were accessible while only 2 were inaccessible) (Fig 4D). In contrast, JQ-1 treatment drastically altered the chromatin accessibility landscape (significant changes of 11,434 ATAC-seq peaks, 7679 of which were accessible while 3,755 were inaccessible) (Fig 4E). Strikingly, combination of JQ-1 treatment and SARS-CoV-2 infection induced 2.2-fold increased change to the accessibility of genomic regions when compared to JQ-1 treatment alone (significant changes in 25,306 ATAC-seq peaks, whereby 17,137 were accessible while 8,169 were inaccessible) (Fig 4F), implying that changes induced by JQ-1 treatment are quantitatively superior to those induced by SARS-CoV-2 infection, which is also illustrated by the low degree of modulation of chromatin accessibility induced by the infection in the context of JQ-1 treatment (Fig 4G).

As expected [50,51], compared to uninfected cells, pathways that drive innate immune responses were enriched in the accessible peaks from SARS-CoV-2-infected cells (S2B Fig). Accessible peaks from JQ-1-treated cells showed no significant association with any particular biological pathway when compared to DMSO-treated cells (S2B and S2C Fig), suggesting that JQ-1 induced more of a global change rather than alterations of specific biological processes. On the other hand, when compared to infected cells, accessible peaks from infected cells in the presence of JQ-1 were mostly enriched with ribosome biogenesis and RNA processing pathways (S2B Fig), including RNA splicing reported to be inhibited by SARS-CoV-2 [52]. Of note, nonsense-mediated mRNA decay, a cellular RNA surveillance pathway that exhibits a broad antiviral activity [53,54], was among the highly enriched pathways in the accessible peaks (S2B Fig). Conversely, pathways associated with sensory perception of smell were downregulated in the accessible peaks from SARS-CoV-2-infected cells, both in the presence of DMSO and JQ-1 (S2C Fig), but not by JQ-1 *per se*, suggesting an inability of JQ-1 to antagonise SARS-CoV-2-mediated downregulation of the smell receptor signalling pathway. Furthermore, accessible peaks from JQ-1-treated cells in the presence of infection were associated with downregulation of the neuropeptide signalling pathway (S2C Fig), as opposed to SARS-CoV-2 infection or JQ-1 treatment alone, suggesting JQ-1’s potential to interfere with neuronal communication specifically in the context of infection. Together, these data show that SARS-CoV-2- and JQ-1-mediated modulation of the chromatin accessibility landscape occurs at multiple levels, with differing magnitudes and breadth.

We next searched for transcription factor (TF) binding motifs that were significantly enriched in the accessible ATAC-seq peaks using the DREME algorithm, which is designed to find short and multiple nonredundant binding motifs of eukaryotic TFs and calculate their statistical significance [55]. In infected cells, accessible peaks were enriched with binding motifs for inflammatory TF families, including Signal Transducer and Activator of Transcription (STAT), Interferon Regulatory Factors (IRF) and NFκB (Fig 4H and S1A Table); which drive IFN signalling and induction of pro-inflammatory cytokines, respectively [56]. This suggests increased accessibility of STAT-, IRF- and NFκB-binding sites to induce SARS-CoV-2-mediated elevated IFN responses and production of pro-inflammatory cytokines [1,19]. Unlike in SARS-CoV-2 infection, the accessible peaks in JQ-1-treated cells showed significant enrichment with several binding motifs of a wide variety of TF families, suggesting a broader modulation of gene expression by JQ-1 over SARS-CoV-2 infection (S1B–S1D Table). Independent of infection, among many others, peaks from JQ-1-treated cells were enriched with binding motifs for TF families such as cap ‘n’ collar basic leucine zipper (CNC-bZIP) and nuclear factor Y (NF-Y) (Fig 4I–4J and S1B–S1D Table), which induce antiviral cellular cytoprotective responses [49,57] and more accessible chromatin [58,59], respectively. The enrichment of CNC-bZIP TF motif in JQ-1 uninfected/JQ-1 infected contrast suggests an inability of SARS-CoV-2 to revert JQ-1-mediated accessibility of this motif (Fig 4K). Detection of these enrichments, independent of SARS-CoV-2 infection, argues for a dominant role of JQ-1 treatment on transcription modulation via TFs belonging to the CNC-bZIP family. Secondly, motifs annotated to a superfamily of steroid-induced nuclear receptor (NR) TFs, whose signalling drives cellular RNA splicing processes [60,61], were also enriched in JQ-1-treated groups irrespective of the infection status (S1B–S1D Table). However, JQ-1 treatment in the presence of SARS-CoV-2 infection (S1C and S1D Table) displayed significant enrichment with TF binding motifs for a wide range of TF families compared to the absence of infection (S1B Table), suggesting that SARS-CoV-2 infection modulates JQ-1-mediated TF profile and likely fine-tunes JQ-1-mediated transcriptome. Interestingly, compared to infected JQ-1-treated cells, peaks from uninfected JQ-1-treated cells were significantly enriched with a binding motif for NFκB-1/NFκB-2 TFs (S1B Table), despite JQ-1 being an inhibitor of the NFκB-1-mediated canonical pathway that induces cytokine production [4]. Together, these data illustrate the genome-wide TF binding profiles that drive the transcriptional programs governing cellular responses to SARS-CoV-2 infection and JQ-1 administration.

### SARS-CoV-2 and JQ-1-mediated modulations of the chromatin accessibility landscape underlie changes in transcriptomic and proteomic profiles

Next, we subjected the samples to bulk RNA-seq and mass spectrometry to establish their transcriptomic and proteomic profiles, respectively, including the downstream effects of SARS-CoV-2- and JQ-1-mediated modulations on the chromatin accessibility landscape. PCA of the RNA-seq data revealed that the samples clustered according to their experimental groups, reflecting similar transcriptomic profiles between replicates in each group (S3A Fig). As in ATAC-seq (S2A Fig), JQ-1 treatment induced more drastic changes than SARS-CoV-2 infection. Samples from naïve and uninfected DMSO-treated cells clustered close to each other, reflecting minimal effect of DMSO treatment on the cellular transcriptome (S3A Fig).

**Fig 5:**
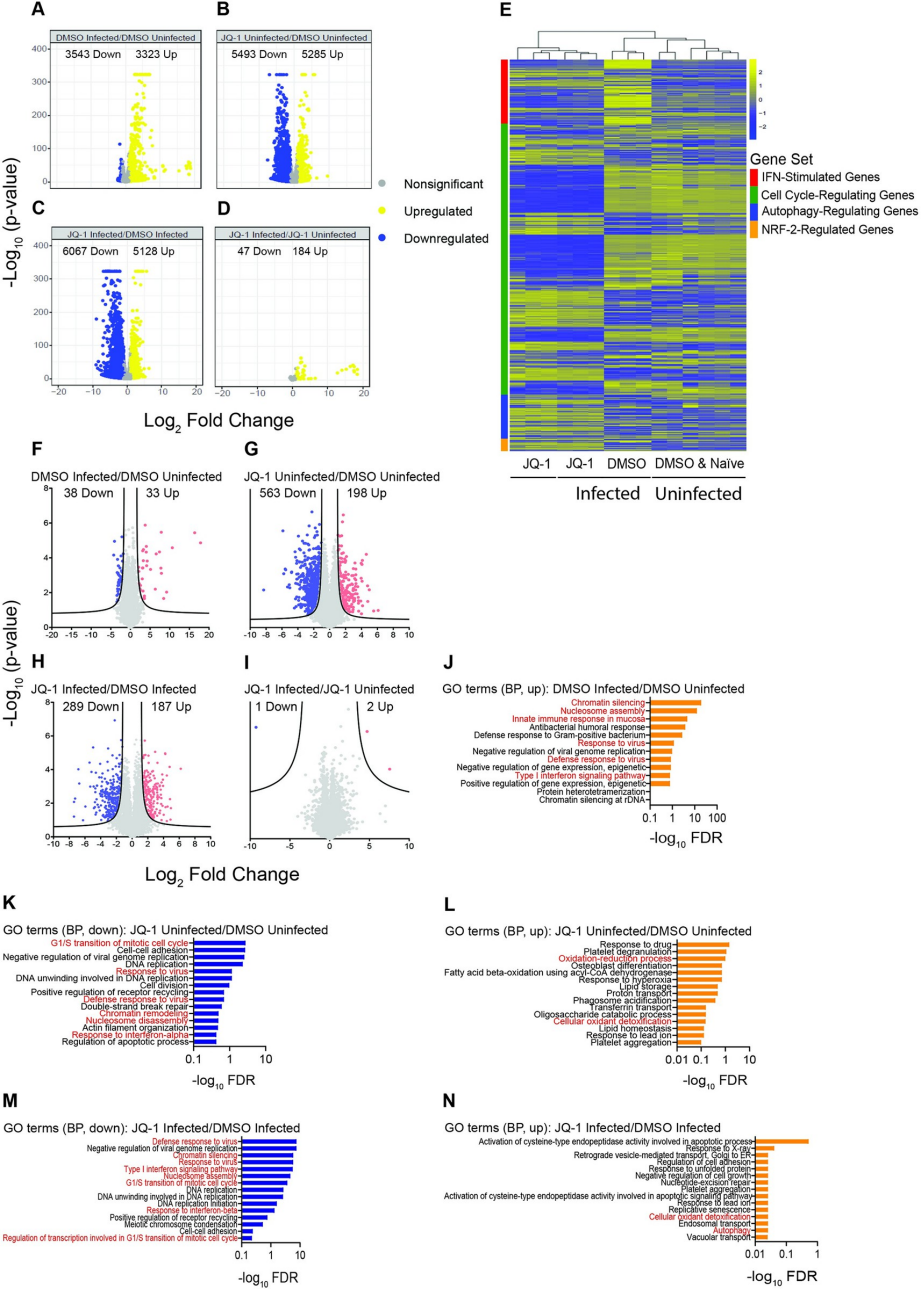
SARS-CoV-2 and JQ-1-mediated modulations of the chromatin accessibility landscape underlie changes in transcriptomic and proteomic profiles. (**A-D**) Volcano plots showing relative log_2_FC and statistical significance [-log_10_ (p-value)] of differentially regulated genes (DRGs) in (**A**) DMSO-treated infected/DMSO-treated uninfected, (**B**) JQ-1-treated uninfected/DMSO-treated uninfected, (**C**) JQ-1-treated infected/DMSO-treated infected and (**D**) JQ-1-treated infected/JQ-1-treated uninfected contrasts. The numbers of significant (FDR of ≤0.05) DRGs are indicated. (**E**) Heatmap of DRGs across four biological pathways (IFN-stimulated genes, cell cycle-regulating genes, autophagy-regulating genes, and NRF-2-regulated genes) scaled as z-score across rows. (**F-I**) Volcano plots showing relative log_2_FC and statistical significance [-log_10_ (p-value)] of differentially abundant proteins in (**F**) DMSO-treated infected/DMSO-treated uninfected, (**G**) JQ-1-treated uninfected/DMSO-treated uninfected, (**H**) JQ-1-treated infected/DMSO-treated infected and (**I**) JQ-1-treated infected/JQ-1-treated uninfected contrasts. The numbers of significantly (FDR ≤0.05) regulated proteins are indicated. (**J-N**) Top enriched (FDR ≤0.05) GO biological pathway terms of upregulated proteins in (**J**) DMSO-treated infected/DMSO-treated uninfected, and differentially regulated proteins in (**K-L**) JQ-1-treated uninfected/DMSO-treated uninfected and (**M-N**) JQ-1-treated infected/DMSO-treated infected contrasts.

SARS-CoV-2 infection induced significant changes to the expression of 6866 genes, of which 3323 were upregulated and 3543 downregulated (Fig 5A). In the absence of infection, JQ-1 treatment induced significant changes to the expression of 10778 genes, of which 5285 were upregulated and 5493 were downregulated (Fig 5B). On the other hand, when compared to infection only, JQ-1 treatment of infected cells induced significant changes to the expression of 11195 genes, with 5128 upregulated and 6067 downregulated (Fig 5C), while the impact of infection in the context of JQ-1 treatment was minor (Fig 5D). Again, these data suggest that JQ-1 treatment modulates the host transcriptome to a larger extent compared to SARS-CoV-2 infection.

Hierarchical clustering of genes driving selected biological pathways revealed that SARS-CoV-2 infection induced upregulation of ISGs (Fig 5E). JQ-1-treated groups, irrespective of their infection status, shared similar transcriptomic signatures, as did the uninfected DMSO-treated and naïve groups. Compared to other groups, JQ-1 treatment altered the cell cycle transcriptomic profile and upregulated genes driving autophagy and NRF-2-mediated cellular cytoprotective response, irrespective of the infection status (Fig 5E). As expected, SARS-CoV-2 induced an upregulation of innate immune signalling pathways in DMSO-treated cells, while pathways driving oxidative phosphorylation (OXPHOS) were among the top downregulated pathways (S3B Fig). In JQ-1-treated cells, SARS-CoV-2 retained its ability to induce innate immune signalling pathways albeit to a lower extent compared to DMSO-treated cells, as indicated by reduced gene ratios, and lost the ability to downregulate the OXPHOS pathways. Conversely, JQ-1 treatment in the presence of infection upregulated the pathways driving OXPHOS and downregulated the innate immune signalling pathways (S3B Fig). However, our analysis is unable to differentiate whether the cellular response is incapacitated or whether there was not enough infection-related pathogen-associated molecular patterns to trigger those responses in the first place. These JQ-1-driven changes were less pronounced or even absent in the absence of infection (S3B Fig). Nucleosome assembly, organisation and other pathways associated with regulation of gene transcription were equally enriched across the analysed contrasts (S3B Fig).

To generate proteomic profiles, we resolved proteins from the whole cell lysate on linear SDS-PAGE gels, visualised the proteins with Bio-Safe Coomassie Blue Stain (S3C Fig) and subjected them to liquid chromatography coupled with tandem mass spectrometry (LC-MS/MS) as described elsewhere [21]. PCA from the proteomic data demonstrated sample clustering according to their experimental groups, reflecting similar proteomic profiles between individual samples in each group (S3D Fig). SARS-CoV-2 infection induced differential abundance of 71 proteins, with 33 upregulated and 38 downregulated (Fig 5F). In the absence of infection, JQ-1 treatment induced differential regulation of 761 proteins, 189 of which were upregulated and 563 downregulated (Fig 5G). JQ-1 treatment in the presence of SARS-CoV-2 infection induced differential regulation of 476 proteins, with 187 upregulated and 289 downregulated (Fig 5H), while the impact of infection in the context of JQ-1 treatment was negligible (Fig 5I).

GO term analysis of the biological processes revealed that innate immune signalling pathways driven by the STATs, IRFs, and NF-κB-1 transcription factors were among the upregulated pathways in SARS-CoV-2-infected samples, as compared to uninfected samples (Fig 5J). Furthermore, chromatin silencing and nucleosome assembly were the top two highly-enriched pathways. Irrespective of the infection status, IFN signalling, chromatin silencing, nucleosome assembly, and G1/S transition of cell cycle were among the downregulated pathways in JQ-1-treated groups (Fig 5K, 5M). Paradoxically, nucleosome assembly and chromatin organisation-related pathways were upregulated at the transcriptomic level by JQ-1 treatment in the presence of infection (S3B Fig), but downregulated at the proteomic level (Fig 5K, 5M). On the other hand, irrespective of the infection status, the NRF-2-driven oxidant detoxification pathway that forms part of the cellular antiviral cytoprotective response was among the upregulated pathways in the JQ-1-treated groups (Fig 5L, 5N). Moreover, the autophagy pathway, which is reduced by SARS-CoV-2 [62], was upregulated in the JQ-1-treated group in the presence of infection (Fig 5N). Together, the transcriptomic and proteomic changes largely recapitulate the modulated biological pathways, which are driven by transcription factors whose binding motifs are enriched as determined by the ATAC-seq data.

### JQ-1 pretreatment antagonises SARS-CoV-2-mediated suppression of the antiviral NRF-2-mediated cytoprotective response

**Fig 6:**
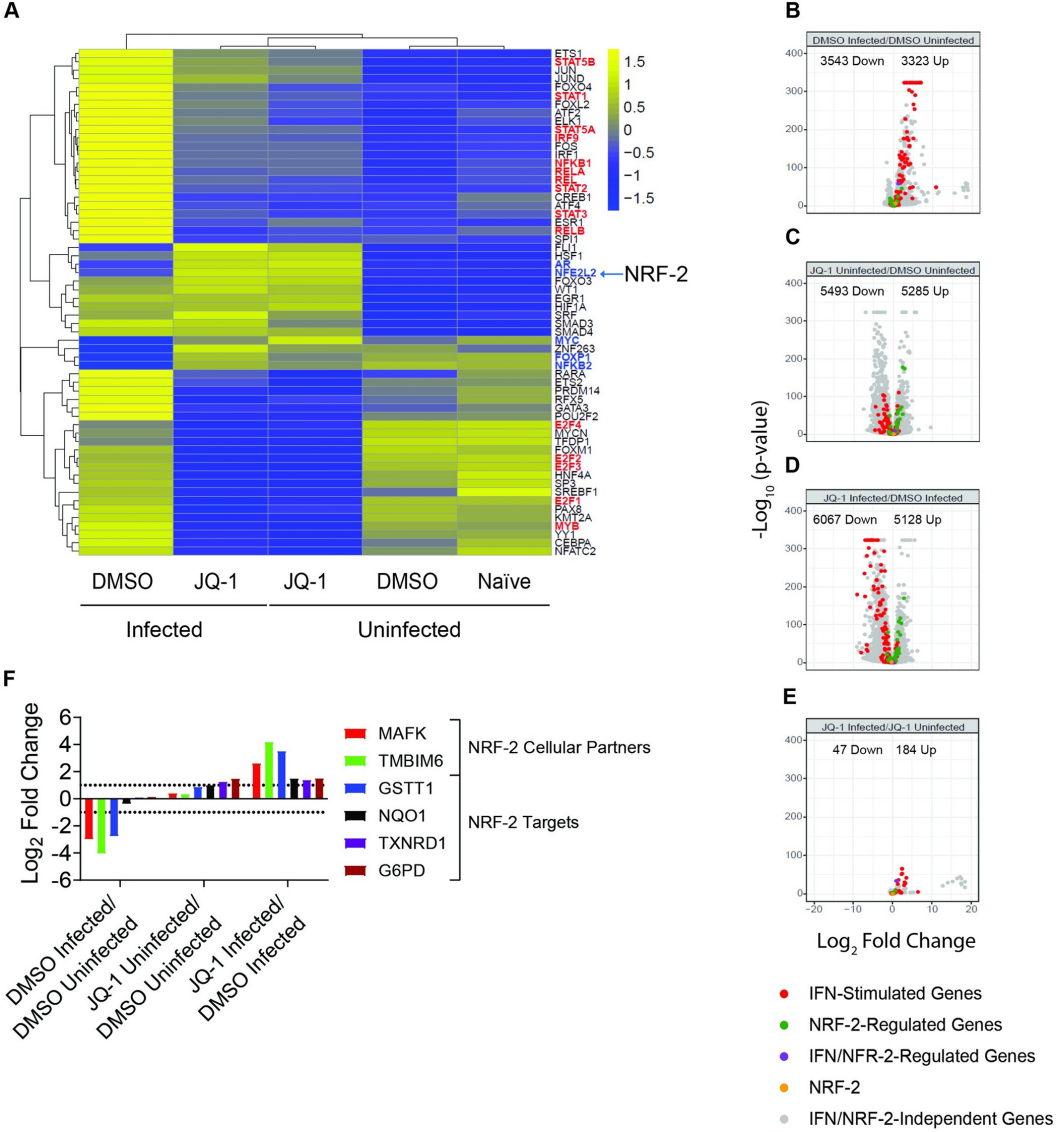
JQ-1 pretreatment antagonises SARS-CoV-2-mediated suppression of the antiviral NRF-2-mediated cytoprotective response. (**A**) Heatmap of scaled TF activity scores in indicated experimental groups from the RNA-seq data, based on the Dorothea database. The highlighted TFs are described in the text, where the ones highlighted in red are induced by SARS-CoV-2 infection and those highlighted in blue induced JQ-1 administration. (**B-E**) Volcano plots showing relative log_2_FC and statistical significance [-log_10_ (p-value)] of differentially expressed genes involved in IFN and NRF-2 signalling in (**B**) DMSO-treated infected/DMSO-treated uninfected, (**C**) JQ-1-treated uninfected/DMSO-treated uninfected, (**D**) JQ-1-treated infected/DMSO-treated infected and (**E**) JQ-1-treated infected/JQ-1-treated uninfected contrasts. (**F**) Log_2_FC analysis of differentially abundant proteins implicated in NRF-2 signalling. The bars indicate relative log_2_FC in protein abundance between indicated contrasts and proteins with a relative log_2_FC of >1 (dashed line) and an FDR≤0.05 were considered significant.

Despite the growing literature on the anti-SARS-CoV-2 activity of JQ-1 and other iBETs [2–5], TFs orchestrating transcriptional responses in the context of JQ-1-mediated anti-SARS-CoV-2 activity remain to be investigated. For this purpose, we performed global TF activity profiling using the bulk RNA-seq dataset, which was collected in parallel with ATAC-seq data to identify TFs with significantly regulated gene module scores across our experimental groups. SARS-CoV-2 infection of Calu-3 cells induced activity of the inflammatory TF families (STATs, IRFs, and NFκB-1) (Fig 6A), whose signalling is associated with severe COVID-19 cases [63]. JQ-1 treatment in the presence of SARS-CoV-2 infection suppressed the activity of these inflammatory TF families, suggesting an anti-inflammatory effect of JQ-1 (Fig 6A). Interestingly, while SARS-CoV-2 infection induced NFκB-1-driven canonical signalling pathway, it strongly suppressed NFκB-2-driven noncanonical signalling pathway, reflecting a more precise SARS-CoV-2-mediated regulation of NFκB signalling pathways.

Irrespective of the infection status, JQ-1 treatment suppressed activity of the TF families that regulate cell cycle and proliferation (E2F & MYB), whose synergistic signalling is associated with severe lung injury in COVID-19 cases [63]. This is in line with JQ-1-mediated dysregulation of cell cycle-regulating genes (Fig 5E) and downregulation of pathways driving G1/S transition of the mitotic cell cycle captured in our proteomic data (Fig 5K, 5M). Furthermore, JQ-1 also induced the transcriptional activities of NRF-2 and androgen receptor (AR), which drive a cellular cytoprotective response [57] and RNA alternative splicing [64,65], respectively (Fig 6A). It is noteworthy that pharmacological induction of NRF-2 signalling by 4-OI and DMF inhibits SARS-CoV-2 replication *in vitro* [49]. NRF-2 and AR belong to CNC-bZIP and steroid nuclear receptor (NR) families of TFs, respectively, whose binding motifs were significantly enriched in the accessible ATAC-seq peaks from JQ-1-treated cells independent of infection (S1B and S1C Table). Together, these data suggest that the chromatin regulatory regions captured in our ATAC-seq data were not only accessible, but also transcriptionally active.

To discern between SARS-CoV-2-mediated upregulation of IFN signalling, inhibition of NRF-2 signalling, and JQ-1-mediated antagonistic effects in these processes, we generated volcano plots to analyse the log_2_FCs of STAT/IRF and NRF-2 target genes. As expected, ISGs were upregulated in the context of SARS-CoV-2 infection (Fig 6B). SARS-CoV-2 infection showed minimal changes on the expression of NRF-2 target genes and no effect on NRF-2 expression *per se* (Fig 6B). JQ-1 treatment induced the downregulation of ISGs in the absence (Fig 6C) and presence (Figs 6D and S5) of SARS-CoV-2 infection. Irrespective of the infection status, JQ-1 treatment induced an upregulation of the expression of NRF-2 target genes (Fig 6C and 6D). Like SARS-CoV-2 infection (Fig 6B), JQ-1 treatment did not significantly change expression of NRF-2 itself, independent of the infection status (Fig 6C and 6D). SARS-CoV-2 infection in the context of JQ-1 treatment imposed minor changes (Fig 6E). These data suggest that both SARS-CoV-2 and JQ-1-mediated modulations of NRF-2 signalling do not significantly alter NRF-2 expression *per se*, but likely modulate the expression of its signalling cofactors.

NRF-2 induces gene expression by binding to the *cis*-acting antioxidant response elements (ARE) on the promoters of its target genes as a heterodimer with the small musculoaponeurotic fibrosarcoma (sMAF) transcription factors, which act as its indispensable cofactors [66,67]. Furthermore, transmembrane B cell lymphoma 2-associated X protein (BAX) inhibitor motif-containing 6 (TMBIM6) protects the host against oxidative stress by inducing NRF-2 signalling [68,69]. Interestingly, the proteomic data showed significant reduction of log_2_FCs for MAFK and TMIBM6, and NRF-2 target proteins in SARS-CoV-2-infected Calu-3 cells (Fig 6F). JQ-1 treatment antagonised these SARS-CoV-2-mediated changes, with more pronounced inhibition in the context of infection (Fig 6F). These data suggest that SARS-CoV-2 suppresses NRF-2 signalling by downregulating its indispensable signalling cofactors.

Further log_2_FC analysis revealed that SARS-CoV-2 infection induced a dramatic upregulation of COQ6 expression (S4 Fig), which drives the production of coenzyme Q10 to shuttle electrons between complexes I, II and III; and associated dehydrogenase enzymes in the mitochondrial electron transport chain (ETC) [70,71]. These data hint at the need for active ubiquinone biosynthetic pathway in SARS-CoV-2 pathogenesis to serve a yet to be identified function other than driving the OXPHOS, which is impaired by SARS-CoV infection (S3B Fig). Moreover, SARS-CoV-2 infection downregulated COX16 that serves as an indispensable component of cytochrome-c-oxidase (complex IV) in the electron transport chain [72] and upregulated the chromatin silencing histone variants (H2AFY, H2AFV and H2AFZ) (S4 Fig), which likely drive the SARS-CoV-2-mediated upregulation of chromatin silencing and nucleosome assembly pathways (Fig 5J). More importantly, SARS-CoV-2 downregulated the expression of YTHDF2, a methyl-6-adenosine (m6A) reader of the RNA molecules in the host, which binds to and degrade m6A-bearing host [73] and viral [74–76] RNA molecules to prevent the expression of aberrant cellular RNA and inhibit viral replication, respectively. JQ-1 displayed an antagonistic effect against the above-mentioned SARS-CoV-2-mediated proteomic modulations (S4 Fig).

### NRF-2 signalling pathway is an integral component of JQ-1-mediated anti-SARS-CoV-2 activity

**Fig 7:**
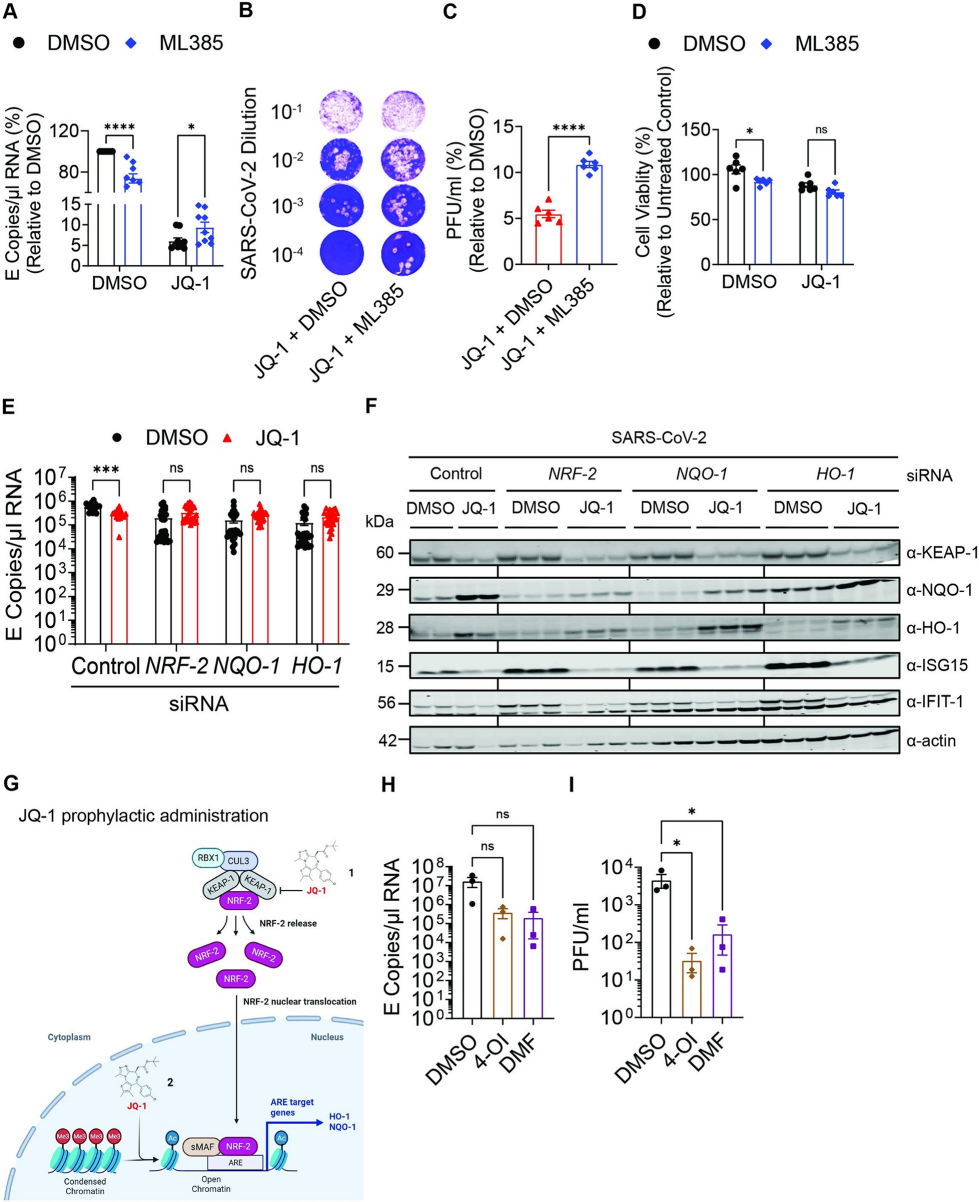
NRF-2 signalling pathway is an integral component of JQ-1-mediated anti-SARS-CoV-2 activity. (**A-C**) Relative quantification of SARS-CoV-2 (**A**) genomic RNA (E copies/μl) and (**B-C**) titers (PFU/ml) in the supernatants harvested at 24 h.p.i. Calu-3 cells were co-treated twice with DMSO; DMSO/ML385 (1.28μM); JQ-1 (2.56μM)/DMSO and JQ-1 (2.56μM/)/ML385 (1.28μM) for 48 hours prior to infection with SARS-CoV-2 (MOI = 0.1) for 24 hours under continuous presence of the drugs. Unpaired parametric t-test with the Holm-Šídák correction for multiple testing was used to compare the means from triplicates of three independent experiments for the viral genomic RNA quantification (E copies/μl). Unpaired parametric t-test was used to compare the means from triplicates of two independent experiments for viral titration (PFU/ml). (**D**) Relative quantification of cell viability in Calu-3 cells co-treated with the drug combinations as mentioned above. Calu-3 cells were co-treated three times every 24 hours for 72 hours and analyzed luminometrically. The graph shows the background-subtracted data normalized to untreated cells. Unpaired parametric t-test with the Holm-Šídák correction for multiple testing was used to compare the means from duplicates of three independent experiments. (**E**) Quantification of SARS-CoV-2 genomic RNA (E copies/μl) in the supernatants of Calu-3 cell knockdowns at 24 h.p.i. Calu-3 cells were siRNA-transfected twice (24 & 72 hours post seeding) as indicated, pretreated twice (72 & 96 hours post seeding) with DMSO or JQ-1 (2.56μM) and infected with SARS-CoV-2 (MOI = 0.1) for 24 hours under continuous presence of the drug. Unpaired parametric t-test with the Holm-Šídák correction for multiple testing was used to compare the means from nine replicates of three independent experiments for targeting siRNAs. Control siRNA had triplicates in n = 1 and six replicates in n = 2 and n = 3. (**F**) Immunoblotting of Calu-3 cell lysates harvested in (**E**) using indicated antibodies. Duplicates and triplicates were loaded for cells transfected with control and targeted siRNAs, respectively. (**G**) Schematic diagram depicting the proposed JQ-1-mediated induction of NRF-2 signalling pathway. Created with www.biorender.com. (**H-I**) Quantification of SARS-CoV-2 (**H**) genomic RNA (E copies/μl) and (**I**) titers (PFU/ml) in the supernatants harvested at 24 h.p.i. Calu-3 cells were pretreated twice for 48 hours with DMSO, 4-OI (125μM) and DMF (125μM) prior to infection with SARS-CoV-2 (MOI = 0.1) for 24 hours under continuous presence of the drug. One-way ANOVA with Dunnett’s multiple comparison test was used to compare the means between experimental groups from three independent experiments. Raw data are shown in **S1 Data**.

Next, we investigated whether pharmacological inhibition of NRF-2 signalling antagonises JQ-1-mediated anti-SARS-CoV-2 activity. Interestingly, co-treatment of Calu-3 cells with JQ-1 and ML385, a specific inhibitor of NRF-2 [77], prior to infection, led to a significant increase in viral RNA copies (Fig 7A) and infectious titers (Fig 7B and 7C) in the supernatant compared to JQ-1- and DMSO-co-treated cells, in the absence of detectable cell toxicity (Fig 7D). Importantly, ML385-mediated rescue of infection was not detected in the absence of JQ-1 treatment, suggesting that ML385 treatment specifically reverted JQ-1-induced NRF-2 signalling (Fig 7A). Furthermore, ML385 displayed some level of toxicity to Calu-3 cells in the context of DMSO co-treatment (Fig 7D), and this could explain ML385-mediated reduction of viral RNA copies in the presence of DMSO (Fig 7A). These data suggest that JQ-1 inhibition of SARS-CoV-2 infection in Calu-3 cells involves, at least partially, efficient NRF-2 signalling. To further address the role of the NRF2-mediated cytoprotective response during JQ-1 treatment, we generated individual Calu-3 cell bulk populations in which we knocked down the expression of NRF-2, NAD(P)H quinone dehydrogenase 1 (NQO-1), heme oxygenase 1 (HO-1) and Kelch-like ECH-associated protein 1 (KEAP-1) by siRNA. Compared to cells transfected with control siRNA, JQ-1´s antiviral activity was absent in cells transfected with siRNAs targeting *NRF2*, *NQO-1* and *HO-1* expression (Fig 7E). We failed to establish a sensitive detection system for NRF-2. However, siRNA knockdown of NQO-1 and HO-1 was confirmed by immunoblotting, and transfection of cells with siRNA targeting NRF-2 was followed by the expected reduction of NQO-1 and HO-1, indirectly suggesting that NRF-2 knockdown occurred. Immunoblotting further revealed that in cells transfected with control siRNA, JQ-1 treatment induced a distinct upregulation of NQO-1 and HO-1, which are two target proteins of NRF-2. These data validate our RNA-seq data, which show that JQ-1 induces NRF-2 signalling pathway without modulating the expression of NRF-2 *per se* (Fig 6C and 6D). Conversely, JQ-1 treatment reduced the abundance of NRF-2 inhibitory protein KEAP-1, potentially resulting in freeing up more of NRF-2 molecules from its inhibitor (KEAP-1) to translocate into the nucleus and switch on the antioxidant responsive elements (AREs), which become more accessible to NRF-2 due to JQ-1-mediated euchromatinization (Fig 7G). Interestingly, the ability of JQ-1 to repress *IFIT1* and *ISG15* expression was preserved in all knock-down conditions, suggesting that the latter and the induction of NRF-2 responses are mechanistically uncoupled. Finally, iBET-independent induction of NRF-2 by 4-OI and DMF resulted in reduced SARS-CoV-2 replication (Fig 7H and 7I), which was particularly visible when measuring the viral titers by plaque assays (Fig 7I). This is in agreement with other reports [49]. Together, these data demonstrate that induction of NRF-2-mediated cytoprotective response is one of the antiviral components induced by JQ-1 treatment.

### SARS-CoV-2 subverts JQ-1-mediated antiviral activity

**Fig 8:**
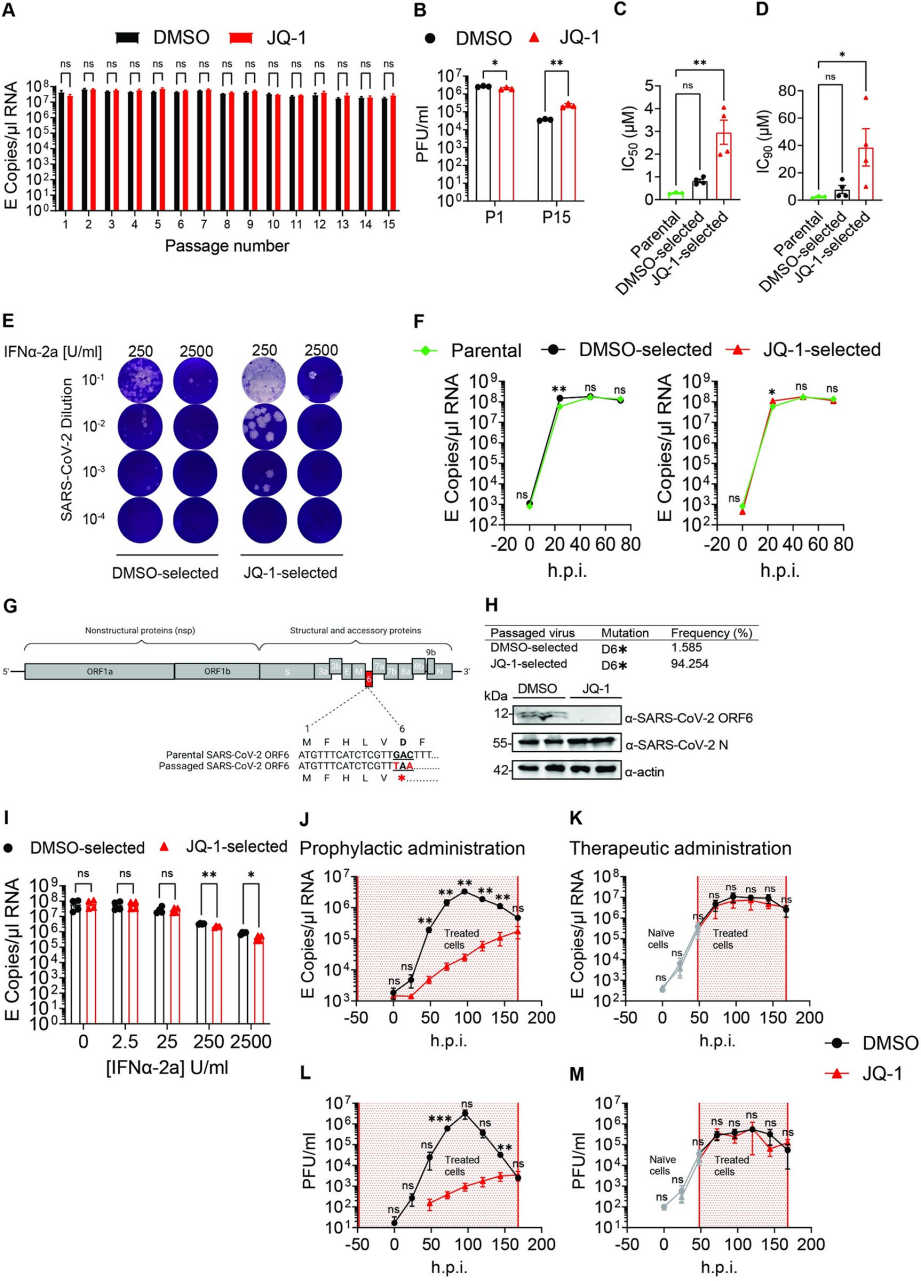
SARS-CoV-2 subverts JQ-1-mediated antiviral activity. (**A**) Quantification of SARS-CoV-2 RNA (E copies/μl) from 15 serial passages under two-fold escalating concentrations of JQ-1, with DMSO as a mock. Unpaired parametric t-test with the Holm-Šídák correction for multiple testing was used to compare the means from triplicates of one experiment. (**B**) Quantification of SARS-CoV-2 titers (PFU/ml) in triplicates from P1 (JQ-1 0.32μM) and P15 (JQ-1 5.12μM) passages under JQ-1 selection. Unpaired parametric t-test with the Holm-Šídák correction for multiple testing was used to compare the means from triplicates of one experiment. Quantification of (**C**) IC_50_ and (**D**) IC_90_ values from JQ-1 dose-dependent inhibition curves determined in JQ-1-treated Calu-3 cells infected with parental and passaged SARS-CoV-2 virions (MOI = 0.1) based on viral RNA concentrations in the supernatant (**S6C Fig**). One-way ANOVA with Dunnett’s multiple comparison test was used to compare the means from three independent experiments with the parental virus and four independent experiments with passaged SARS-CoV-2 virions. (**E**) Representative plaque phenotypes derived in Vero E6 cells showing plaque morphologies from passaged SARS-CoV-2 virions following infection of IFN-treated Calu-3 cells. (**F**) Viral growth kinetics in DMSO-treated Calu-3 cells showing viral RNA quantities (E copies/μl) from parental and passaged (P15) SARS-CoV-2 virions (MOI = 0.1). Unpaired parametric t-test with the Holm-Šídák correction for multiple testing was used to compare the means from four independent experiments. (**G**) Schematic diagram of the SARS-CoV-2 genome depicting the premature stop codon at position six of ORF6 in sequences derived from the passaged (P15) virions. (**H**) Quantification of ORF6 D6* mutation frequency from sequences generated from the genome of passaged (P15) SARS-CoV-2 virions. Immunoblot analysis of SARS-CoV-2-ORF6 and nucleocapsid (N) expression in Calu-3 cells infected with DMSO-selected and JQ-1-selected SARS-CoV-2 (P15, MOI = 0.1). (**I**) Quantification of SARS-CoV-2 genomic RNA (E copies/μl) from passaged (P15) SARS-CoV-2 virions in the supernatants of IFN-treated Calu-3 cells at 24 h.p.i. Calu-3 cells were pretreated for 24 hours with indicated concentrations of IFNα-2a prior to infection with SARS-CoV-2 under continuous IFN treatment. Unpaired parametric t-test with the Holm-Šídák correction for multiple testing was used to compare the means from duplicates of two independent experiments. (**J-K**) Virus growth kinetics in DMSO and JQ-1 (2.56 μM)-treated hBAECs depicting SARS-CoV-2 RNA quantities (E copies/μl) in the culture supernatants following (**J**) prophylactic and (**K**) therapeutic drug administration. (**L-M**) Virus growth kinetics in DMSO and JQ-1 (2.56 μM)-treated hBAECs depicting SARS-CoV-2 infectious titers (PFU/ml) in the culture supernatants following following (**L**) prophylactic and (**M**) therapeutic drug administration. The area shaded in red indicates the time period of drug administration and the grey lines in the context of therapeutic drug administration indicate infection before drug administration. Unpaired parametric t-test with the Holm-Šídák correction for multiple testing was used to compare the means from triplicates of one experiment. Raw data are shown in **S1 Data**.

SARS-CoV-2 can adapt to and acquire resistance against potent virus-directed antivirals such as remdesivir (RDV) *in vitro* [78,79], a replication bottleneck that induces the emergence of RDV-resistant SARS-CoV-2 variants *in vivo* [80]. To investigate whether SARS-CoV-2 can acquire resistance to iBETs, we serially passaged it in Calu-3 cells under escalating two-fold concentrations of JQ-1 (S6A Fig), along with RDV as a reference. SARS-CoV-2 serial passaging under increasing two-fold concentrations of RDV led to a gradual reduction of viral RNA copies in the supernatant, with a potential resistance phenotype emerging at the earliest at passage 15 (S6B Fig). In contrast, SARS-CoV-2 serial passaging under escalating two-fold concentrations of JQ-1 displayed unrelenting high concentrations of viral RNA copies from passage two until passage 15 (Fig 8A). These data suggest early adaptation of SARS-CoV-2 to JQ-1. Quantification of viral titers from passages one and 15 revealed that SARS-CoV-2 virions were sensitive to JQ-1 treatment at the beginning of the passaging experiments, and acquired resistance to JQ-1 following serial passaging (Fig 8B).

To determine the extent to which SARS-CoV-2 passaging under JQ-1 led to the acquisition of resistance, we investigated the sensitivity of DMSO and JQ-1-selected SARS-CoV-2 virions to JQ-1 in Calu-3 cells by quantifying the viral RNA copies in the supernatant and calculating concentrations required to inhibit 50% (IC_50_) and 90% (IC_90_) of viral replication. Compared to parental SARS-CoV-2, only virions that were serially passaged under JQ-1 exhibited a significant reduction in sensitivity to JQ-1 treatment in Calu-3 cells, but not the DMSO-selected virions (Figs 8C and 8D and S6C and S6D). The JQ-1-selected virus formed much larger plaques compared to the DMSO-selected virus (Fig 8E), suggesting that passaging SARS-CoV-2 under JQ-1 enhances the viral cytopathic effect. Viral growth kinetics in Calu-3 cells showed that DMSO- and JQ-1-selected SARS-CoV-2 virions acquired minimal, if any, replication fitness compared to the parental SARS-CoV-2 virions (Fig 8F). NGS analysis revealed virus sequences encoding a premature stop codon at position six of ORF6, which normally encodes for aspartic acid, in the genomes of passaged SARS-CoV-2 virions (Fig 8G and 8H and S2A and S2B Table). Our analysis further showed that this mutation pre-existed as a minor variant (0.182% mutation frequency) in the genome of the parental virus (S2B Table). This premature stop codon was far more frequently detected in JQ-1-selected virions (94.254% mutation frequency) than DMSO-selected virions (1.585% mutation frequency) (Fig 8H and S2A Table). Of note, no minority variants were detected in SARS-CoV-2 spike (S), arguing against the emergence of a virus expressing a modified S protein with the potential to spread under conditions of low ACE2 expression. Consequently, SARS-CoV-2 selection under JQ-1, but not DMSO, led to the emergence of ORF6-deficient SARS-CoV-2 virions as shown by the immunoblotting (Fig 8H). Interestingly, in a comparative analysis of recombinant isogenic viruses expressing and lacking ORF6, we reproduced reduced sensitivity to JQ-1 in the context of ORF6 deficiency, which was more pronounced in plaque assays than in viral RNA quantification (S6E and S6F Fig). Together, these data suggest that ORF6 deficiency contributes to, or even determines JQ-1 resistance observed in the JQ-1-selected P15 population (Figs 8C and 8D and S6C). As expected for ORF6-deficient SARS-CoV-2 [28,81,82], loss of ORF6 expression sensitised the JQ-1-selected P15 virus population to treatment with exogenous IFN-I (Fig 8I). These data constitute the concept of collateral drug sensitivity and provide the first experimental support, to our knowledge, for evolutionary trap vaccines in the context of COVID-19.

Finally, we performed kinetic and multi-cycle infection experiments in hBAECs to compare JQ-1-mediated anti-SARS-CoV-2 activity in the contexts of prophylactic and therapeutic treatment. In the set-up mimicking prophylactic administration, we started treatment of hBAECs with DMSO or JQ-1 48 hours prior to SARS-CoV-2 infection (S6G Fig). For therapeutic administration, we infected treatment-naïve hBAECs and started treatment 48 hours post-infection (S6H Fig). JQ-1 treatment was continued for the duration of the entire experiment. Prophylactic administration of JQ-1 resulted in antiviral activity that was more pronounced earlier post infection, which however vanished over time, as indicated by the gradual increase in viral RNA copies (Fig 8J) and infectious titers (Fig 8L) in the supernatant. Strikingly, administration of JQ-1 following establishment of infection failed to curb SARS-CoV-2 replication, as indicated by comparable viral RNA copies (Fig 8K) and infectious titers (Fig 8M) in the supernatant samples of DMSO- and JQ-1-treated hBAECs. Together, these data suggest that JQ-1 exhibits a transient antiviral activity, which is subverted by SARS-CoV-2 in the context of a full-blown infection.

## Discussion

Prior studies attributed the iBET-mediated anti-SARS-CoV-2 activity to the downregulation of cellular ACE2 expression [2–4], but failed to capture the pleiotropic nature of the antiviral state induced by these compounds and its susceptibility to SARS-CoV-2 subversion. Here, we show that JQ-1-mediated anti-SARS-CoV-2 activity goes beyond the downregulation of ACE2 expression by inducing a vast transcriptional modulation, including the mounting of antiviral NRF-2-mediated cytoprotective response. Furthermore, we show that JQ-1-mediated antiviral activity occurs transiently when administered prophylactically and nullified by SARS-CoV-2 when administered therapeutically following an established infection, suggesting effective viral antagonistic strategies.

In agreement with prior studies in Calu-3 cells [2–5], iBETs displayed an antiviral activity against SARS-CoV-2 infection in lung epithelial Calu-3 cells and hBAECs when administered prophylactically, suggesting that the iBET-mediated anti-SARS-CoV-2 activity is readily translated from cell lines *in vitro* to clinically relevant human-derived infection models. The antiviral activity of JQ-1 and its analogues has largely been reported in the context of SARS-CoV-2 parental strains [2–4] and a variant-of-concern (VOC) Delta [5]. The resistance of MERS-CoV to JQ-1-mediated inhibition is likely determined, to a large extent, by the ACE2-independent entry of MERS-CoV. Supporting this argument is the selective entry inhibition of the *Sarbecoviruses*—but not MERS-CoV—by knock-out-mediated disruption of the mammalian SWItch/Sucrose Non-Fermentable (mSWI/SNF) complex that regulates ACE2 expression [83]. Interestingly, one of the components making up mSWI/SNF complex is a short isoform of BRD4, which predisposes this chromatin remodeler and its downstream effects to JQ-1-mediated inhibition [84]. This argues in favour of the striking similarities between our data and those reported elsewhere [83], at least in the context of JQ-1-selective inhibition between the *Sarbecoviruses* and MERS-CoV. In addition, since our data show that NRF-2 induction is an integral part of JQ-1-induced antiviral program, it is tempting to speculate that MERS-CoV is resistant to inhibition by the NRF-2-mediated cytoprotective response. One testable hypothesis is that this difference can potentially be attributed to the interactions between SARS-CoV-2-E and cellular BET proteins [5,13], through a conserved histone motif present in SARS-CoV-E and SARS-CoV-2-E proteins [12,85], which is lacking in MERS-CoV-E protein [85].

The regulatory landscape orchestrating the iBET-mediated transcriptional responses to SARS-CoV-2 infection has remained largely unexplored. Accessible peaks in SARS-CoV-2-infected samples were enriched with binding motifs for the inflammatory TF family (STATs, IRFs and NFκB), which were significantly associated with the upregulation of IFN and cytokine signalling pathways. On the other hand, accessible peaks from the same samples were significantly associated with the downregulation of smell receptor signalling pathways, reminiscent of the olfactory dysfunction reported in COVID-19 cases [86–88]. Significant association of accessible peaks from JQ-1-treated samples in the presence of infection with the downregulation of the smell receptor signalling pathways suggests that JQ-1 is unable to dampen SARS-CoV-2-mediated olfactory dysfunction [86–88].

Similar to the ATAC-seq data, we captured massive differential regulation of genes and proteins in JQ-1-treated samples, irrespective of infection, compared to SARS-CoV-2 infection. Upregulated genes and proteins in SARS-CoV-2-infected samples were significantly associated with the upregulation of IFN and cytokine signalling pathways. In line with the detected enrichment of accessible binding motifs for the inflammatory TF family, supervised hierarchical clustering further showed that SARS-CoV-2 infection induced the transcription of ISGs. In accordance with reported SARS-CoV-2-mediated impairment of mitochondrial biogenesis [89], downregulated genes in SARS-CoV-2-infected samples were significantly associated (among others) with the downregulation of pathways driving mitochondrial OXPHOS. The upregulation of chromatin silencing and nucleosome assembly pathways at the proteomic level in SARS-CoV-2-infected samples is consistent with SARS-CoV-2-ORF8-mediated chromatin compaction [90] and may represent the epigenomic mechanism underlying SARS-CoV-2-mediated host-shutoff [91].

Interestingly, despite inducing a strong NFκB-1 TF activity that drives inflammatory cytokine production [19], SARS-CoV-2 infection induced an equally strong suppression of NFκB-2 TF activity, which regulates the maturation of antibody-producing B cells and development of germinal centres (GC) in the lymph nodes [92,93]. Conversely, irrespective of infection, JQ-1 suppressed NFκB-1 TF activity and induced NFκB-2 TF activity. It is noteworthy that inhibition of NFκB-1 transcriptional footprint inhibits SARS-CoV-2 replication [94]. These data reflect a carefully executed and precise regulation of NFκB-mediated signalling pathways by SARS-CoV-2 infection and JQ-1 administration.

Association of the upregulated genes and proteins in JQ-1-treated samples with the downregulation of IFN signalling and viral genome replication pathways validates JQ-1-mediated suppression of innate immune responses [2,4,13] and antiviral activity [2–5]. SARS-CoV-2 infection limits induction of autophagy [62]. Pharmacological induction of NRF-2, which is a cellular cytoprotective response-inducing transcription factor that belongs to the CNC-bZIP TF family, inhibits SARS-CoV-2 replication [49]. Of note, a binding motif for CNC-bZIP TF family was among the top enriched TF motifs in accessible peaks from all JQ-1-treated samples, irrespective of infection.

Of particular interest, supervised hierarchical clustering revealed that JQ-1 treatment upregulated the expression of autophagy-regulating and NRF-2 target genes, irrespective of SARS-CoV-2 infection. In accordance, pathway enrichment analysis at the proteomic level revealed an upregulation of autophagy in JQ-1-treated samples in the presence of infection and NRF-2-mediated cellular oxidant detoxification pathway irrespective of infection status. Motif enrichment for the TFs belonging to CNC-bZIP and NF-Y families in the context of JQ-1 administration, irrespective of infection, revealed the chromatin regulatory landscape underlying JQ-1-mediated induction of NRF-2 signalling [95], which exhibits an anti-SARS-CoV-2 activity in the context of 4-OI and DMF-mediated induction [49]. Moreover, signalling by the NF-Y TFs increases chromatin accessibility by preventing nucleosome encroachment [58,59]. Therefore, enrichment of the binding motifs for the NF-Y TFs supports the highly accessible chromatin landscape detected in the context of JQ-1 treatment. Together, these data show that JQ-1 induced the activities of NRF-2, which drive the induction of an antiviral cellular cytoprotective response [49].

SARS-CoV-2 infection upregulated the ISGs, but displayed minimal alterations on the expression of NRF-2 target genes, without altering NRF-2 expression *per se*. On the other hand, JQ-1 administration downregulated the expression of ISGs and induced the expression of NRF-2 target genes irrespective of infection. However, like SARS-CoV-2 infection, JQ-1 administration also did not alter NRF-2 expression, despite significantly altering the expression of its target genes. These data suggest that differential regulation of NRF-2 signalling by SARS-CoV-2 infection and JQ-1 administration does not alter NRF-2 expression *per se*, but the expression of its target genes.

Accordingly, the proteomic data showed that SARS-CoV-2 infection induced significant log_2_FC downregulation of MAFK and TMBIM6, which act as indispensable cofactors in the induction of NRF-2 signalling [66–69]. Accompanying SARS-CoV-2-mediated downregulation of NRF-2 signalling cofactors was the downregulation of NRF-2 target proteins. JQ-1 administration induced an upregulation of MAFK, TMBIM6, and NRF-2 target proteins, with a more pronounced effect in the presence of infection. These data complement SARS-CoV-2- and JQ-1-mediated differential regulation of NRF-2 TF activity and suggest that both SARS-CoV-2 infection and JQ-1 administration regulate NRF-2 TF activity by modulating the expression of its signalling cofactors and target genes, but not NRF-2 expression *per se*. Suppression of NRF-2-mediated cytoprotective response in biopsies from COVID-19 patients [49] supports the biological plausibility of our data.

4-OI- and DMF-mediated induction of NRF-2-driven cytoprotective response with anti-SARS-CoV-2 activity [49] and known JQ-1-mediated induction of NRF-2 signalling [95], prompted us to pursue the effect of JQ-1-mediated induction of NRF-2 signalling in the context of JQ-1-mediated anti-SARS-CoV-2 activity. Partial antagonism of JQ-1-mediated anti-SARS-CoV-2 activity by ML385, a specific inhibitor of NRF-2 [77], and siRNA-mediated knockdown of NRF-2 and its target genes (*NQO-1* and *HO-1*) suggest that induction of NRF-2 signalling by JQ-1 administration contributes to JQ-1-mediated anti-SARS-CoV-2 activity. This is consistent with NRF-2-mediated anti-SARS-CoV-2 activity reported in the context of 4-OI- and DMF-mediated induction of NRF-2 [49]. The combination of JQ-1-mediated downregulation of ACE2 expression and induction of NRF-2 signalling pathway shown in our study, suggest that JQ-1 exhibits a pleiotropic anti-SARS-CoV-2 activity that affects multiple steps of the viral replication cycle.

How exactly JQ-1-mediated induction of NRF-2 signalling exerts an anti-SARS-CoV-2 activity remains unknown. A previous study [49] proposed that the induction of hypoxia-inducible-factor-1-alpha (HIF-1α) gene expression program induced by 4-OI administration, which was downregulated in biopsies from COVID-19 patients along with NRF-2 signalling pathway, potentially contribute to NRF-2-mediated antiviral cytoprotective response. In contrast, another study [96] reported that SARS-CoV-2-mediated induction of ROS stabilises HIF-1α to induce its gene expression program that promotes glycolysis to sustain SARS-CoV-2 replication in monocytes. Consistent with the later report, we found induction of HIF-1α TF activity following SARS-CoV-2 infection in Calu-3 cells.

Furthermore, irrespective of infection, JQ-1 administration also induced HIF-1α TF activity. Downregulation of HIF-1α TF activity in control groups suggest that both SARS-CoV-2 infection and JQ-1 administration induce HIF-1α signalling. This is consistent with the idea of an alternative JQ-1-driven HIF-1α TF activity-inducing mechanisms that are independent of SARS-CoV-2-mediated ROS production and argues against the potential effect of HIF-1α-mediated signalling in NRF-2-induced anti-SARS-CoV-2 activity proposed elsewhere [49]. Alternatively, NRF-2 signalling induces the expression of HO-1 [49,97], which catalyses the catabolism of heme into biliverdin, iron, and carbon monoxide (CO) [98]. Interestingly, the products of HO-1-mediated heme catabolism exhibit a broad-spectrum antiviral activity [99], and as expected, our data show that siRNA-mediated knockdown of HO-1 in Calu-3 cells abolishes JQ-1-mediated anti-SARS-CoV-2 activity. Moreover, we found that even NQO-1 knockdown abolished JQ-1-mediated anti-SARS-CoV-2 activity. This suggests that JQ-1-mediated anti-SARS-CoV-2 activity requires an intact NRF-2 signalling pathway to induce a multifaceted antiviral program, which is mediated at least through more than one of its target proteins.

Of particular interest, prophylactic administration of free iron and biliverdin to Vero E6 cells prior to infection inhibited SARS-CoV-2 replication [100]. In contrast, despite binding to SARS-CoV-2-S protein with nanomolar affinity and dampening S protein interactions with neutralising antibodies, administration of 100 μM biliverdin at the time of infection did not inhibit infection of Vero E6 cells by SARS-CoV-2 [101]. These studies [100,101] suggest that like JQ-1, biliverdin-induced anti-SARS-CoV-2 activity is directed at the host and not the infecting virions. This is consistent with the maintenance of infection efficiency by the virions secreted under JQ-1 treatment. Taken together, these data shed light on the mechanism underlying NRF-2-mediated anti-SARS-CoV-2 activity, which still remains to be fully unravelled.

Immune selection of SARS-CoV-2 has led to the emergence of VOCs bearing an arsenal of mutations in the S protein and other viral proteins, which confer immune evasion capabilities and transmission superiority [102,103]. SARS-CoV-2 adaptation to virus-directed antivirals such as RDV has been reported *in vitro* [78,79] and *in vivo* [80]. Here, as early as passage one (P1) under JQ-1 selection, SARS-CoV-2 RNA copies increased in the supernatant and reached a plateau that was maintained across 15 passages despite the subsequent two-fold escalations of JQ-1 concentrations. Only JQ-1-selected SARS-CoV-2 virions showed a significant reduction of sensitivity to JQ-1 treatment and induced the formation of large plaques. These data suggest that passaging SARS-CoV-2 under JQ-1 induced the emergence of SARS-CoV-2 variants with reduced sensitivity to JQ-1 and enhanced cytopathic effect. Unlike the acquisition of resistance to RDV that led to viral replication defect *in vitro* [78,79], acquisition of resistance to JQ-1 by JQ-1-selected SARS-CoV-2 virions did not come at the expense of the viral replication fitness.

NGS analysis of SARS-CoV-2 virions passaged under JQ-1 treatment revealed acquisition of a premature stop codon at position six of ORF6 (94.254% mutation frequency), where aspartic acid (D) from the parental virus was substituted with a stop codon to constitute an ORF6^D6STOP^ mutation. This mutation was less frequent in DMSO-selected virions (1.585% mutation frequency). Further analysis revealed that this mutation was not introduced by passaging experiments, as it existed in the parental virus albeit as a minor variant with a mutation frequency of 0.182%. The exponential increase of this mutation following SARS-CoV-2 passaging under JQ-1 compared to DMSO suggests that JQ-1 creates a cellular environment that favours an evolutionary trade-off of ORF6.

Like JQ-1, SARS-CoV-2-ORF6 antagonises IFN signalling [28,81,82]. This IFN signalling-repressive effect of JQ-1 may have minimised the need for ORF6-mediated repression of IFN signalling and promoted ORF6 trade-offs. This is consistent with the early emergence of SARS-CoV-2 variant harbouring an in-frame deletion in ORF6 following passaging in IFN-deficient Vero E6 cells [104]. In contrast, replication of virions passaged under DMSO are expected to benefit from preserving the ORF6-mediated antagonism of IFN signalling. In agreement, we found that this ORF6 evolutionary trade-off sensitised JQ-1-selected SARS-CoV-2 virions to IFN-I treatment in Calu-3 cells. This constitutes collateral drug sensitivity and provides evidence that JQ-1, and likely other iBETs, can be used to steer SARS-CoV-2 virions towards IFN evolutionary traps to facilitate their immune clearance rather than as sole candidates against COVID-19.

The accessory proteins of SARS-CoV-2, including ORF6, modulate viral pathogenicity. Infection of k18-hACE2 mice with a recombinant SARS-CoV-2 virus lacking ORF6 induced a delayed weight loss recovery compared to infection with recombinant SARS-CoV-2 virions lacking other accessory proteins, suggesting that ORF6-deficiency enhances SARS-CoV-2 virulence [105]. This is in line with the enhanced cytopathic effect displayed by JQ-1-selected SARS-CoV-2 virions, which are also ORF6-deficient, through formation of large plaques. Acquisition of aggressive virulence by viruses following evolutionary trade-offs of their innate immune antagonists is necessary to compensate for their increased susceptibility to immune clearance by destroying the host before mounting effective immune responses. Our data obtained in the context of the recombinant SARS-CoV-2 virions validate that loss of ORF6 expression is associated, and maybe even driving resistance to JQ-1-mediated antiviral activity. Future work is required, however, to illuminate the molecular basis underpinning this acquired resistance to JQ-1-mediated antiviral activity in the context of ORF6-deficiency.

The accumulating evidence from our study and previous studies [2–5] show that iBETs exhibit an anti-SARS-CoV-2 activity when administered prophylactically and not therapeutically [13]. However, our data further show that the iBET-mediated anti-SARS-CoV-2 activity in the context of prophylactic administration is transient and susceptible to viral subversion. Whether SARS-CoV-2 infection counteracts JQ-1-mediated antiviral activity or acquires alternative capabilities to infect and replicate in the host remains to be addressed in future studies. Interestingly, in one study [13], administration of iBETs at the time of infection, as opposed to pretreatment, resulted in absence of an inhibitory effect and even exacerbation of SARS-CoV-2 replication. In line with this report, JQ-1 displayed no antiviral activity when administered in a therapeutic set-up in our study. The proviral effect of therapeutically administered JQ-1 reported elsewhere [13], which is absent in our study, may be related to different time points of sample harvest following infection (48 versus 24 hours, respectively). Since SARS-CoV-2 infection potently suppresses NRF-2-mediated antiviral cytoprotective responses [49], subsequent and long-term JQ-1 administration may be unable to revert this effect, resulting in SARS-CoV-2-mediated nullification of JQ-1-induced anti-SARS-CoV-2 activity. JQ-1 and SARS-CoV-2 exert diametrically opposed impacts on the NRF-2 pathway, and the viral effect is, on the long run, dominant over JQ-1´s effect. More work is required to specifically understand how exactly infection interferes with the maintenance of JQ-1-induced antiviral state.

Together, these data evoke questions about the clinical suitability for both prophylactic and therapeutic administration of JQ-1 (and likely other iBETs) in the context of COVID-19 and illuminate the potential hurdles that iBETs will have to overcome in order to improve disease prognosis.

## Supporting information

S1 Fig Prophylactic administration of iBETs inhibits SARS-CoV-2 infection. (**A**) Dose response curves (n = 3) showing the effect of the indicated iBETs on the viability of Calu-3 cells. Calu-3 cells were treated three times every 24 hours for 72 hours and analysed luminometrically. The data were background-subtracted and normalised to untreated cells. The graph shows the data from iBET-treated cells relative to DMSO-treated cells. (**B**) Relative quantification of viability in DMSO and JQ-1 (2.56 μM)-treated hBAECs. Cultures were treated three times every 24 hours for 72 hours and analysed luminometrically. The graph shows the background-subtracted data normalised to untreated cells. Unpaired parametric t-test was used to compare the means from duplicates of two independent experiments. (**C-D**) Dose response curves (n = 3) showing the effect of the indicated iBETs on the quantities of (**C**) SARS-CoV-2 genomic RNA (E copies/μl) and (**D**) infectious titers (PFU/ml) in the supernatant at 24 h.p.i. Calu-3 cells were pretreated twice for 48 hours prior to infection with SARS-CoV-2 (MOI = 0.1) for 24 hours under continuous presence of the drug. It is noteworthy that the data points do not properly fit the curve to enable robust IC_50_/IC_90_ calculations, and so the presented figures should be interpreted with caution. (**E**) Dose response curves (n = 3) showing the effect of the indicated iBETs on the viability of Calu-3 cells. Calu-3 cells were treated three times every 24 hours for 72 hours and analysed luminometrically. The data were background-subtracted and normalised to untreated cells. The graph shows the data from iBET-treated cells relative to DMSO-treated cells. Raw data are shown in **S1 Data**.
(PDF)

S2 Fig SARS-CoV-2 and JQ-1-mediated modulations of the chromatin accessibility landscape modulate biological pathways. (**A**) PCA of significantly accessible ATAC-seq peaks showing the variance of peak accessibility profiles between experimental groups. Each symbol represents a technical replicate (**B-C**) Dot plots of GO biological pathway terms from genes annotated to the ATAC-seq peaks with significantly (**B**) increased and (**C**) decreased accessibilities between indicated contrasts. Dot colour represents normalised enrichment score (NES) and dot size (peak ratio) represents the number of significant peaks related to the GO biological pathway term relative to the total number of significant peaks.
(PDF)

S3 Fig SARS-CoV-2 infection and JQ-1 treatment modulate the transcriptomic and proteomic profiles. (**A**) PCA of significantly expressed genes showing the variance of gene expression profiles between experimental groups. Each symbol represents a technical replicate (**B**) Dot plot of GO biological pathway terms showing pathway enrichment from differentially expressed genes (DRGs) between indicated contrasts. Pathways were selected by filtering for the top 15 pathways with the largest (absolute value) normalised enrichment score (NES) per contrast. (**C**) Bio-Safe Coomassie Blue staining of proteins from Calu-3 cell lysates resolved on linear (7.5%) SDS-PAGE gels prior to mass spectrometry analysis. (**D**) PCA of significantly abundant proteins showing the variance of protein profiles between experimental groups. Each symbol represents a technical replica.
(PDF)

S4 Fig SARS-CoV-2 infection modulates abundance of proteins involved in oxidative phosphorylation, chromatin condensation, and epitranscriptome. **(A)** Log_2_FC analysis of differentially abundant proteins implicated in oxidative phosphorylation (COQ6 and COX16), chromatin silencing (H2AFY, H2AFV & H2AFZ), and epitranscriptomics (YTHDF2) in indicated contrasts. The bars indicate relative log_2_FC in protein abundance between experimental groups in each contrast, and proteins with a relative log_2_FC of 1 and an FDR≤0.05 were considered significant.
(PDF)

S5 Fig Prophylactic administration of JQ-1 suppresses the expression of *IFIT1* and *MX2* mRNA levels. (**A-B**) Quantification of the dose-dependent effect of DMSO and JQ-1 on the mRNA expression levels of (**A**) *IFIT1* and (**B**) *MX2* in Calu-3 cells. Calu-3 cells were pretreated for 48 hours with corresponding concentrations of DMSO or JQ-1 (0.04–5.12 μM) prior to infection with SARS-CoV-2 (MOI = 0.1) for 24 hours under continuous treatment. At post infection, infected cells were RNA-extracted and analysed by qRT-PCR for mRNA expression from the indicated genes. Unpaired parametric t-test with the Holm-Šídák correction for multiple testing was used to compare the means from three independent experiments.
(PDF)

S6 Fig ORF6 deficiency confers resistance to JQ-1 inhibition and SARS-CoV-2 subverts JQ-1-mediated antiviral activity in kinetic infection assays. (**A**) Schematic diagram of SARS-CoV-2 passaging in Calu-3 cells under two-fold escalating concentrations (0.32, 0.64, 1.28, 2.56, and 5.12 μM) of JQ-1. Each drug concentration was kept constant for two passages before escalation by two-fold for the next two passages. Samples were taken at 48 h.p.i. until passage ten, the point where the highest non-toxic concentration of JQ-1 (5.12 μM) was reached. Serial passaging continued for the next five passages under a constant concentration of JQ-1 (5.12 μM), during which samples were taken at 72 h.p.i. Created with www.biorender.com. (**B**) Quantification of SARS-CoV-2 RNA (E copies/μl) from 15 serial passages under two-fold escalating concentrations of remdesivir, with DMSO as a mock. Unpaired parametric t-test with the Holm-Šídák correction for multiple testing was used to compare the means from quadruplicates of one experiment. (**C**) Dose response curves (parental n = 3, passaged virions n = 4) showing the dose-dependent effect of JQ-1 on the quantities of parental and passaged (P15) SARS-CoV-2 genomic RNA (E copies/μl) in the supernatant at 24 h.p.i. Calu-3 cells were pretreated twice for 48 hours prior to infection with parental and passaged (P15) SARS-CoV-2 virions (MOI = 0.1) for 24 hours under continuous presence of the drug. (**D**) Quantification of resistance phenotypes of passaged (P15) SARS-CoV-2 virions to JQ-1 treatment. Fold resistance is presented as the IC_50_ concentrations of passaged (P15) SARS-CoV-2 virions relative to IC_50_ concentration of the parental virus. (**E-F**) Dose response curves showing the dose-dependent effect of JQ-1 on the quantities of (**E**) recombinant SARS-CoV-2 genomic RNA (E copies/μl) and (**F**) infectious titers (PFU/ml) in the supernatant at 24 h.p.i. Calu-3 cells were pretreated twice for 48 hours prior to infection with indicated recombinant viruses (MOI = 0.1) for 24 hours under continuous presence of the drug. Data originate from triplicates of four independent experiments in genomic RNA (E copies/μl) and four independent experiments in infectious titers (PFU/ml). (**G-H**) Schematic diagrams of SARS-CoV-2 growth kinetics in hBAECs in the presence of DMSO or JQ-1 (2.56 μM). Created with www.biorender.com. Cultures were pretreated twice for 48 hours with a drug, followed by SARS-CoV-2 (2x10^4^ PFUs) infection and drug administration to constitute a (**G**) prophylactic administration. Cultures were followed for 168 hours, during which supernatants were taken every 24 hours followed by fresh drug administration. To constitute a (**H**) therapeutic administration, treatment-naïve hBAECs were infected with SARS-CoV-2 (2x10^4^ PFUs) for 48 hours to establish production infection, during which supernatants were taken every 24 hours. At 48 h.p.i., cultures were subjected to drug administration and followed until 168 h.p.i. as mentioned above. Raw data are shown in **S1 Data**.
(PDF)

S1 Table Enrichment of transcription factor binding motifs in accessible ATAC-seq peaks in (**A**) DMSO-treated infected/DMSO-treated uninfected, (**B**) JQ-1-treated uninfected/DMSO-treated uninfected, (**C**) JQ-1-treated infected/DMSO-treated infected and (**D**) JQ-1-treated infected/JQ-1-treated uninfected contrasts. The motif search was conducted using the DREME algorithm to annotate the motifs to the known transcription factor families. The height of the letter represents the frequency of each base in the motif.
(PDF)

S2 Table Codons mapped to SARS-CoV-2 ORF6 between positions 27217–27219 in the genome of (**A**) input SARS-CoV-2 and (**B**) serially passaged (P15) SARS-CoV-2 virions under increasing concentrations of DMSO and JQ-1 in Calu-3 cells. The stop codon is marked with an asterisk.
(PDF)

S1 Data Raw data corresponding to the figure panels showing normalizations. (XLSX)
