# Supplementary figures and images for: Pharmacological inhibition of bromodomain and extra-terminal proteins induces an NRF-2-mediated antiviral state that is subverted by SARS-CoV-2 infection
Source: PLoS Pathog. 2023 Sep 25;19(9):e1011657. doi: 10.1371/journal.ppat.1011657 (PMC10629670; doi:10.1371/journal.ppat.1011657)

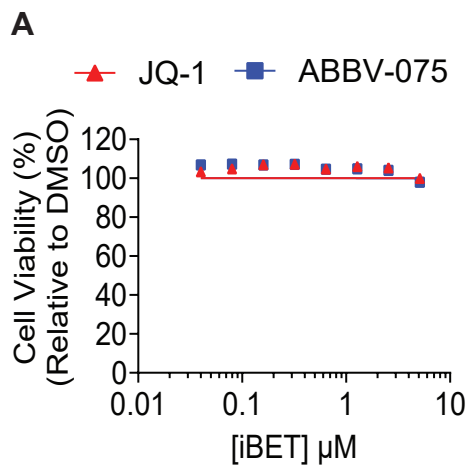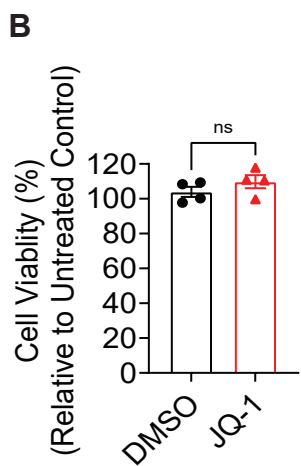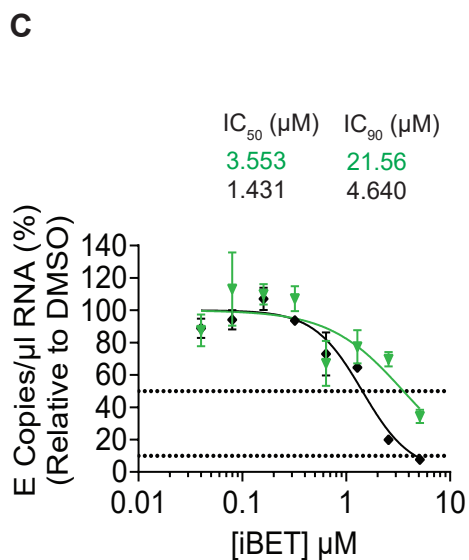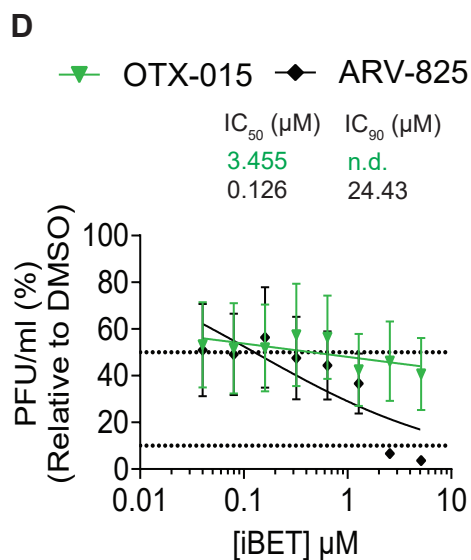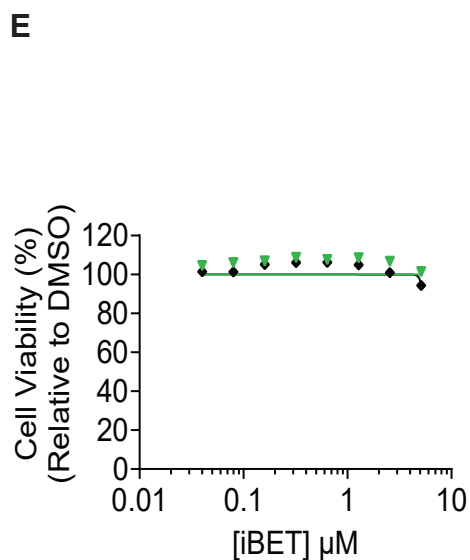

Supplement: S1 Fig — (A) Dose response curves (n = 3) showing the effect of the indicated iBETs on the viability of Calu-3 cells. Calu-3 cells were treated three times every 24 hours for 72 hours and analysed luminometrically. The data were background-subtracted and normalised to untreated cells. The graph shows the data from iBET-treated cells relative to DMSO-treated cells. (B) Relative quantification of viability in DMSO and JQ-1 (2.56 μM)-treated hBAECs. Cultures were treated three times every 24 hours for 72 hours and analysed luminometrically. The graph shows the background-subtracted data normalised to untreated cells. Unpaired parametric t-test was used to compare the means from duplicates of two independent experiments. (C-D) Dose response curves (n = 3) showing the effect of the indicated iBETs on the quantities of (C) SARS-CoV-2 genomic RNA (E copies/μl) and (D) infectious titers (PFU/ml) in the supernatant at 24 h.p.i. Calu-3 cells were pretreated twice for 48 hours prior to infection with SARS-CoV-2 (MOI = 0.1) for 24 hours under continuous presence of the drug. It is noteworthy that the data points do not properly fit the curve to enable robust IC50/IC90 calculations, and so the presented figures should be interpreted with caution. (E) Dose response curves (n = 3) showing the effect of the indicated iBETs on the viability of Calu-3 cells. Calu-3 cells were treated three times every 24 hours for 72 hours and analysed luminometrically. The data were background-subtracted and normalised to untreated cells. The graph shows the data from iBET-treated cells relative to DMSO-treated cells. Raw data are shown in S1 Data. (PDF) [file ppat.1011657.s001.pdf]

A

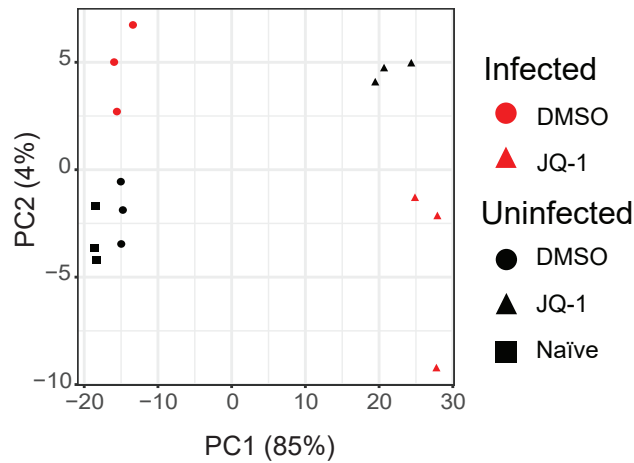

B

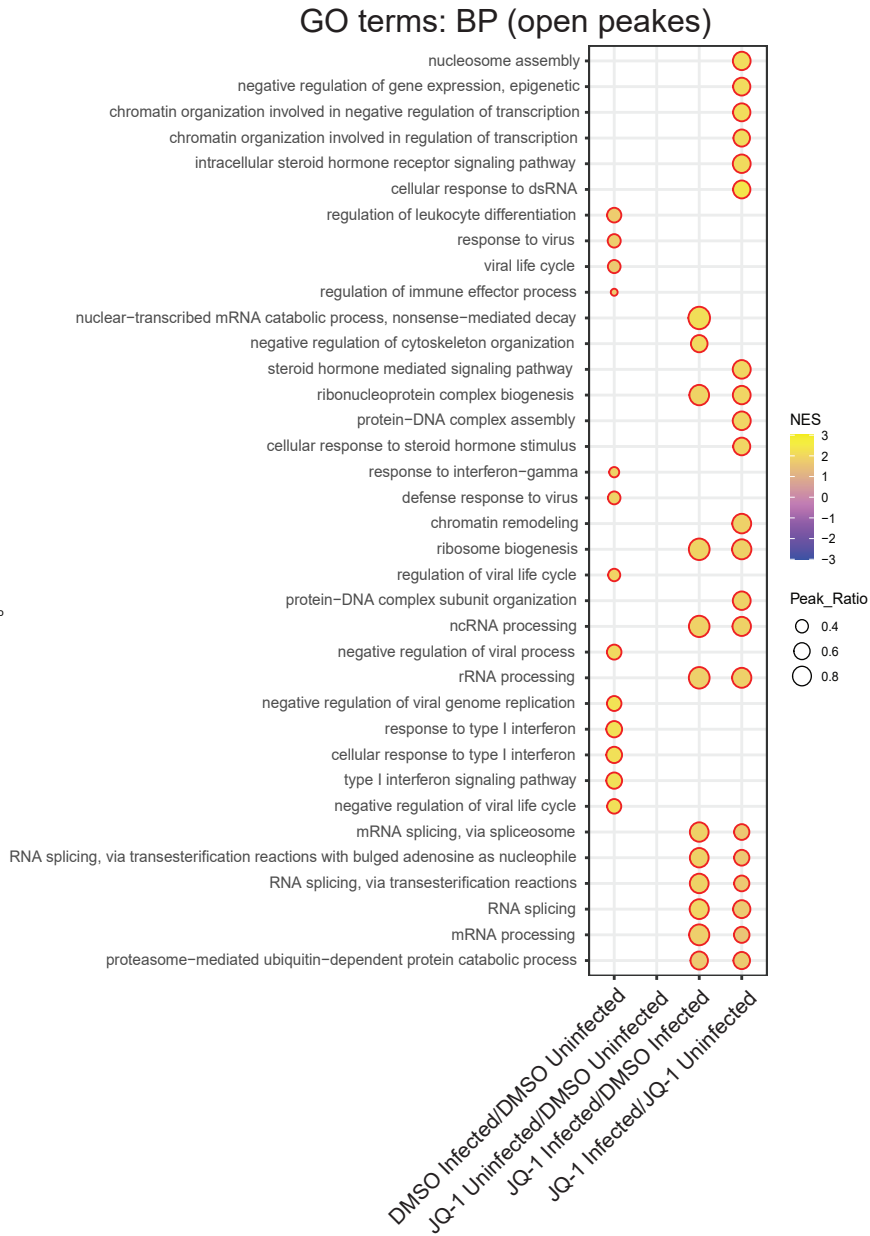

C

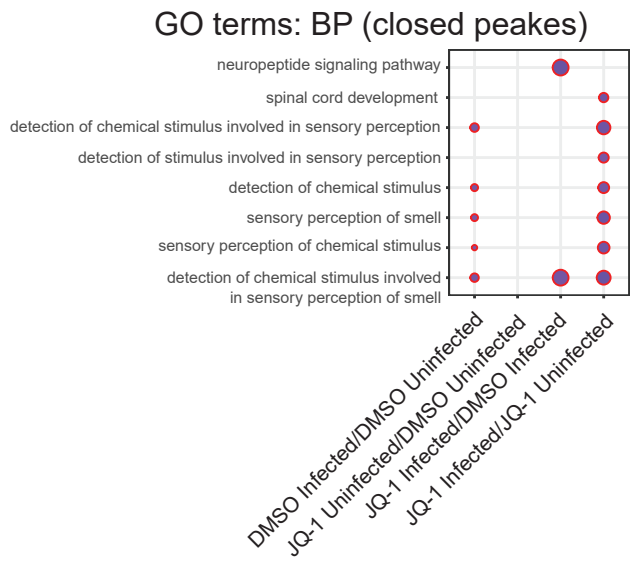

Supplement: S2 Fig — (A) PCA of significantly accessible ATAC-seq peaks showing the variance of peak accessibility profiles between experimental groups. Each symbol represents a technical replicate (B-C) Dot plots of GO biological pathway terms from genes annotated to the ATAC-seq peaks with significantly (B) increased and (C) decreased accessibilities between indicated contrasts. Dot colour represents normalised enrichment score (NES) and dot size (peak ratio) represents the number of significant peaks related to the GO biological pathway term relative to the total number of significant peaks. (PDF) [file ppat.1011657.s002.pdf]

A

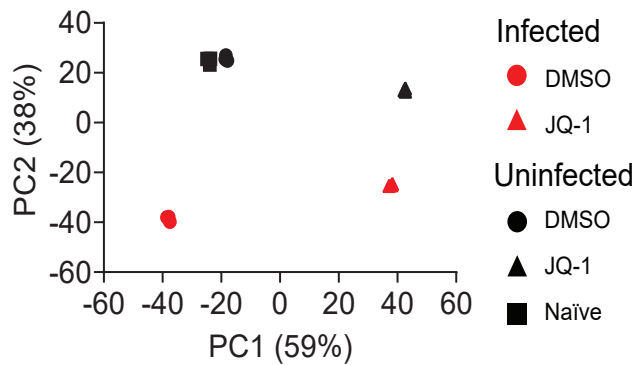

C

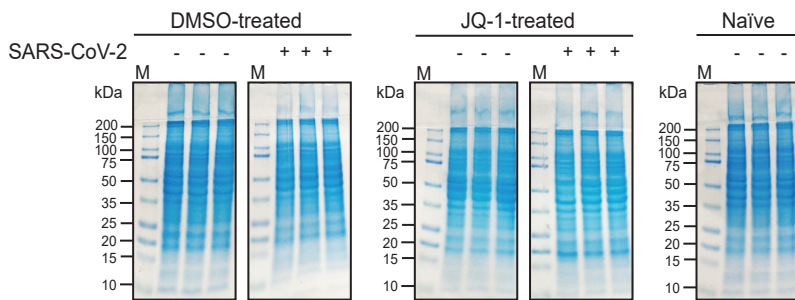

D

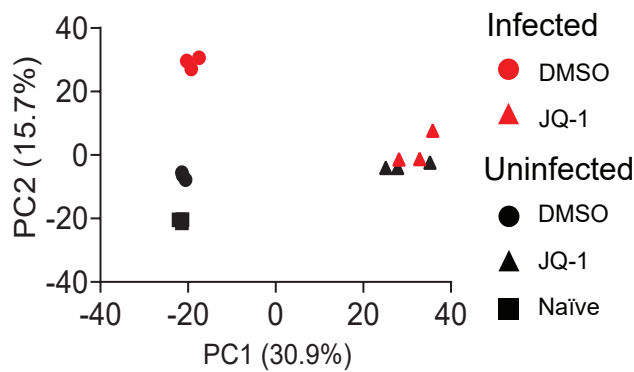

B

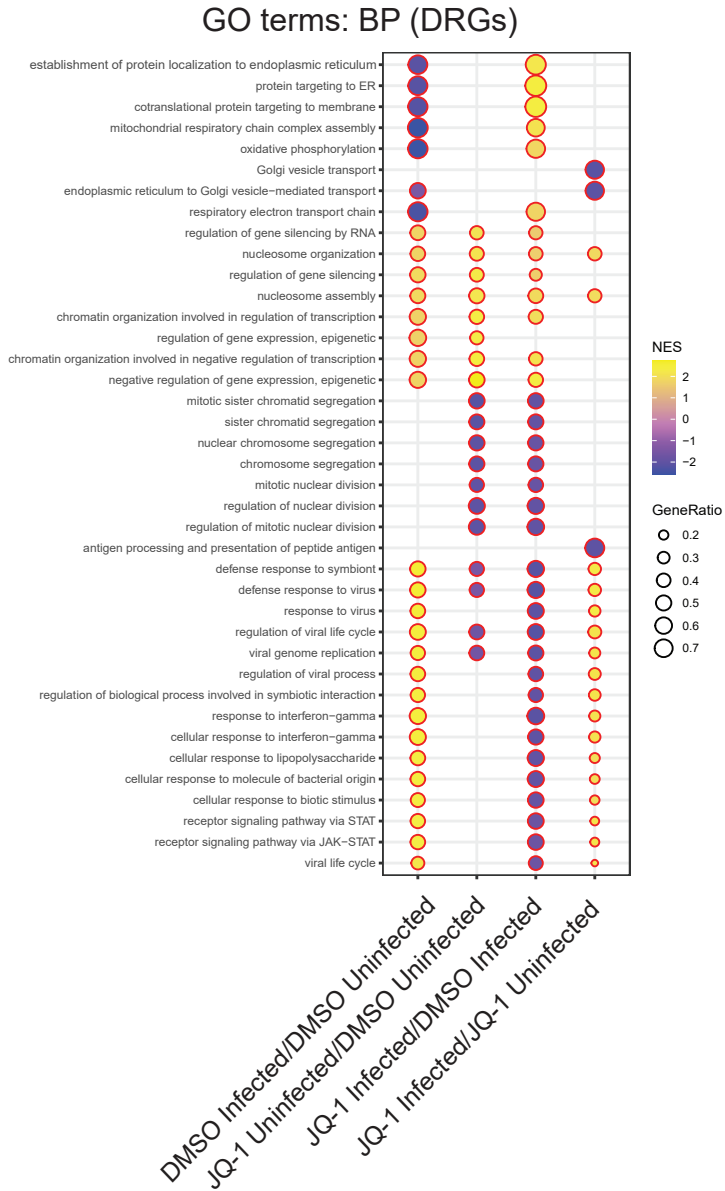

Supplement: S3 Fig — (A) PCA of significantly expressed genes showing the variance of gene expression profiles between experimental groups. Each symbol represents a technical replicate (B) Dot plot of GO biological pathway terms showing pathway enrichment from differentially expressed genes (DRGs) between indicated contrasts. Pathways were selected by filtering for the top 15 pathways with the largest (absolute value) normalised enrichment score (NES) per contrast. (C) Bio-Safe Coomassie Blue staining of proteins from Calu-3 cell lysates resolved on linear (7.5%) SDS-PAGE gels prior to mass spectrometry analysis. (D) PCA of significantly abundant proteins showing the variance of protein profiles between experimental groups. Each symbol represents a technical replica. (PDF) [file ppat.1011657.s003.pdf]

A

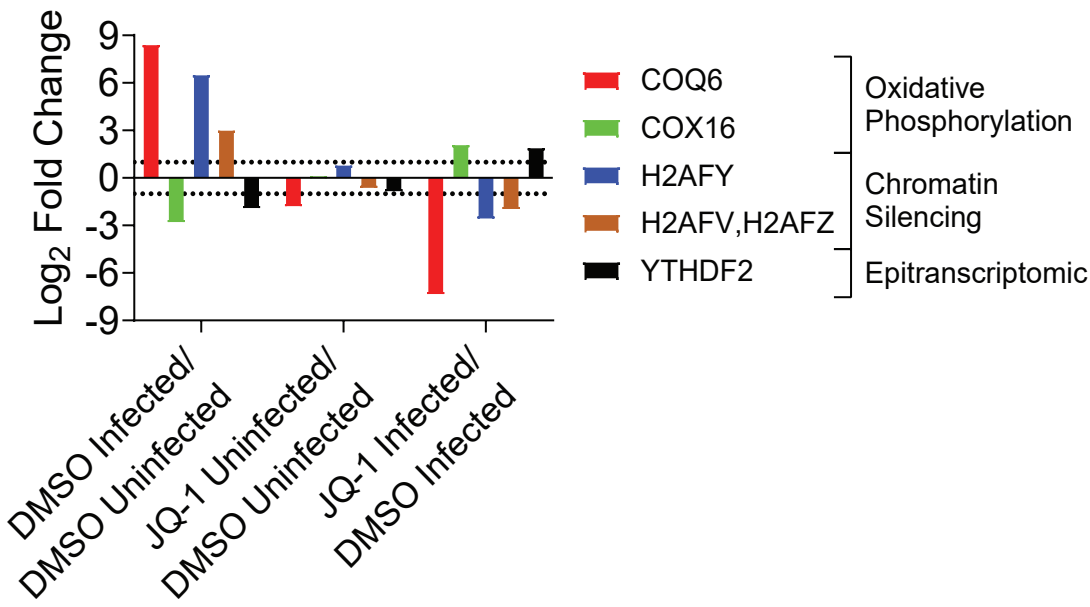

Supplement: S4 Fig — (A) Log2FC analysis of differentially abundant proteins implicated in oxidative phosphorylation (COQ6 and COX16), chromatin silencing (H2AFY, H2AFV & H2AFZ), and epitranscriptomics (YTHDF2) in indicated contrasts. The bars indicate relative log2FC in protein abundance between experimental groups in each contrast, and proteins with a relative log2FC of 1 and an FDR≤0.05 were considered significant. (PDF) [file ppat.1011657.s004.pdf]

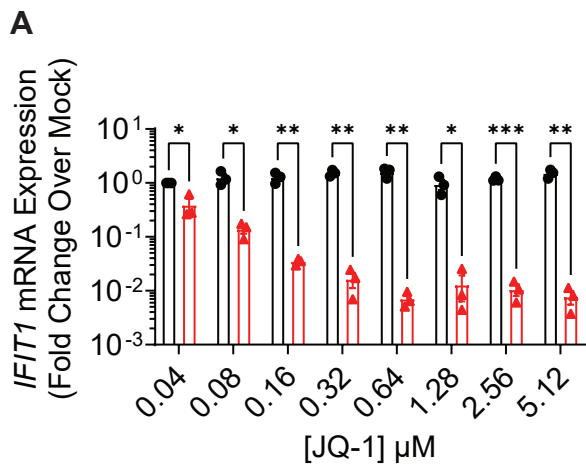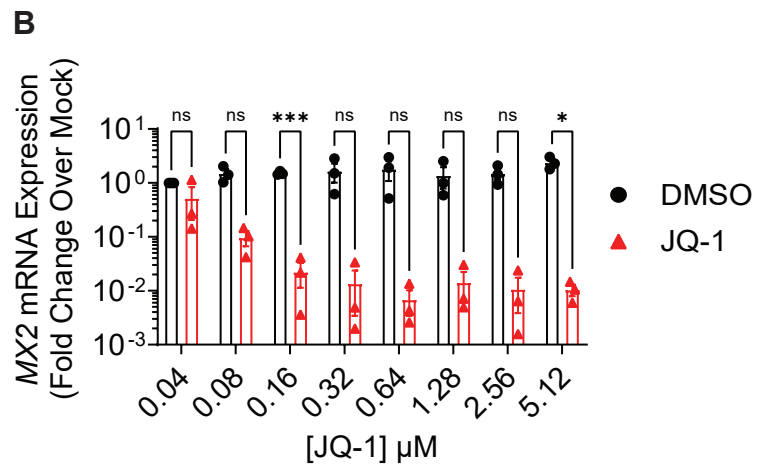

Supplement: S5 Fig — (A-B) Quantification of the dose-dependent effect of DMSO and JQ-1 on the mRNA expression levels of (A) IFIT1 and (B) MX2 in Calu-3 cells. Calu-3 cells were pretreated for 48 hours with corresponding concentrations of DMSO or JQ-1 (0.04–5.12 μM) prior to infection with SARS-CoV-2 (MOI = 0.1) for 24 hours under continuous treatment. At post infection, infected cells were RNA-extracted and analysed by qRT-PCR for mRNA expression from the indicated genes. Unpaired parametric t-test with the Holm-Šídák correction for multiple testing was used to compare the means from three independent experiments. (PDF) [file ppat.1011657.s005.pdf]
