# Supplementary material for: Pharmacological inhibition of bromodomain and extra-terminal proteins induces an NRF-2-mediated antiviral state that is subverted by SARS-CoV-2 infection
Source: PLoS Pathog. 2023 Sep 25;19(9):e1011657. doi: 10.1371/journal.ppat.1011657 (PMC10629670; doi:10.1371/journal.ppat.1011657)

A

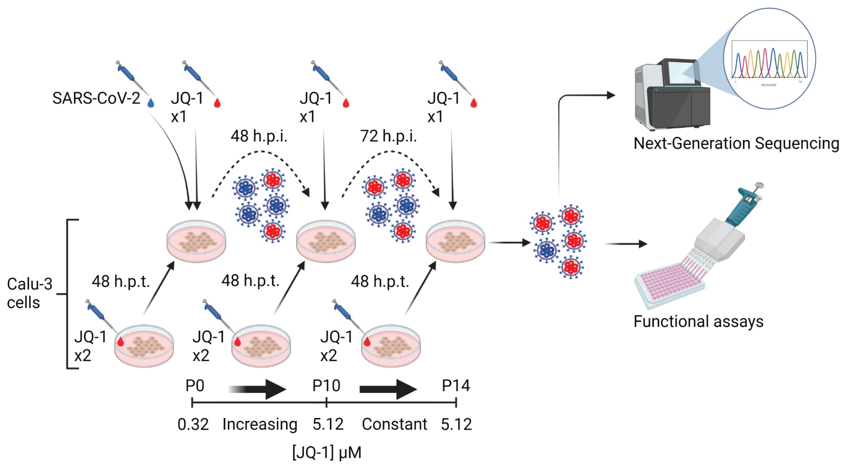

B

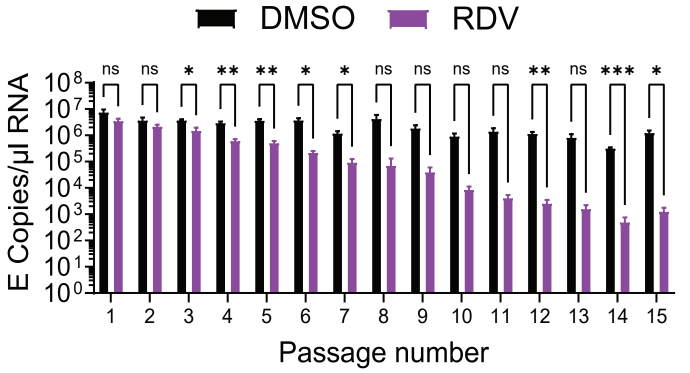

C

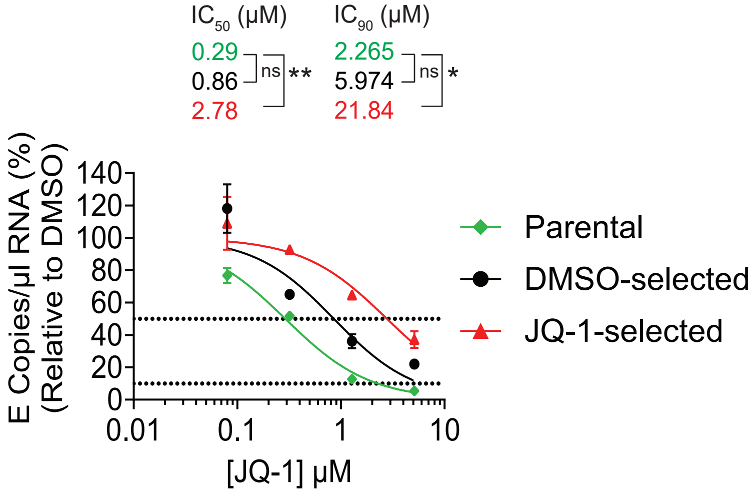

D

Virus	JQ-1	
	IC50 (95% CI)	Fold resistance (relative to Parental)
Parental	0.2905 (0.2329 – 0.3603)	-
DMSO-selected	0.8551 (0.4542 – 1.748)	2.9
JQ-1-selected	2.781 (1.608 – 5.720)	9.6

E

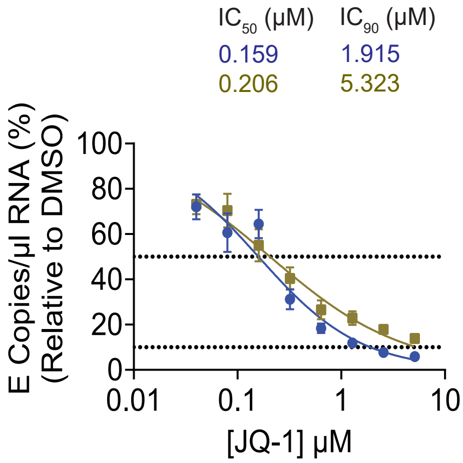

F

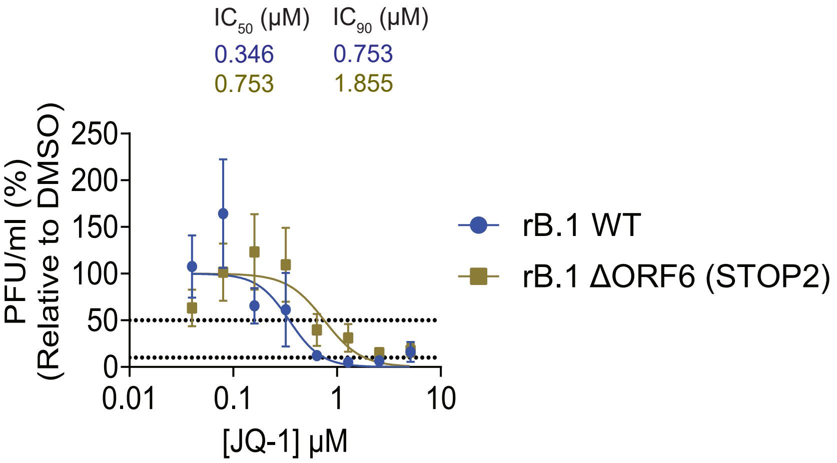

G

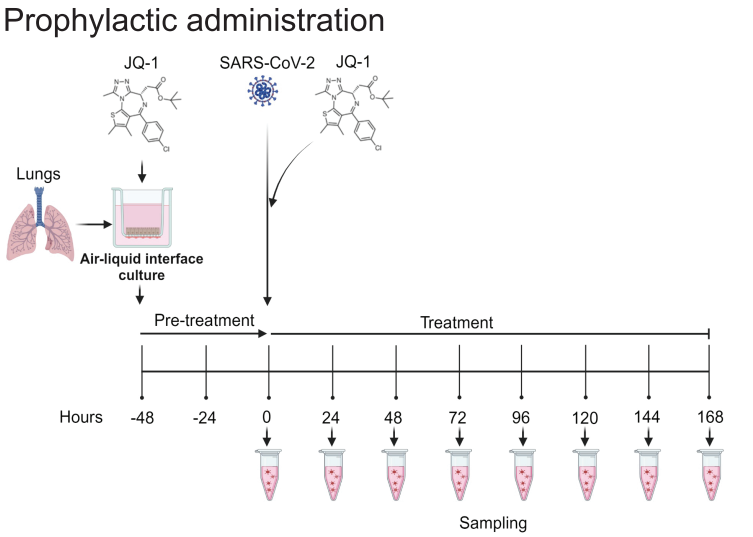

H

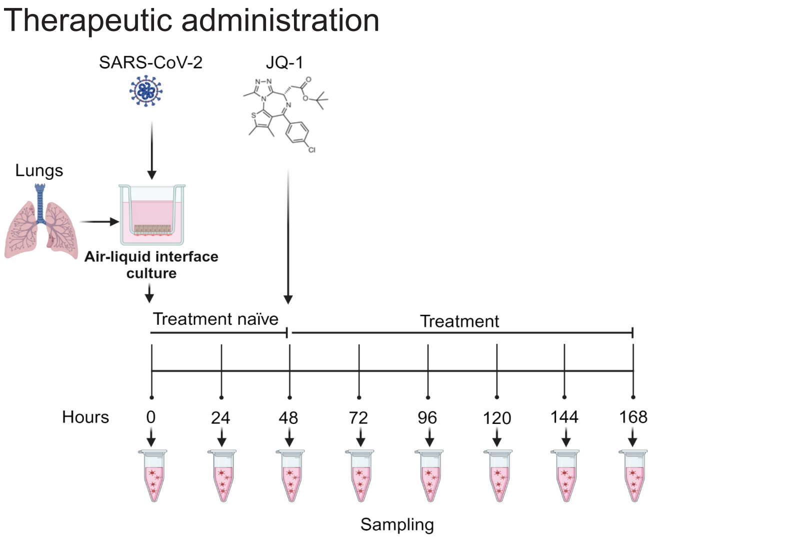

Supplement: S6 Fig — (A) Schematic diagram of SARS-CoV-2 passaging in Calu-3 cells under two-fold escalating concentrations (0.32, 0.64, 1.28, 2.56, and 5.12 μM) of JQ-1. Each drug concentration was kept constant for two passages before escalation by two-fold for the next two passages. Samples were taken at 48 h.p.i. until passage ten, the point where the highest non-toxic concentration of JQ-1 (5.12 μM) was reached. Serial passaging continued for the next five passages under a constant concentration of JQ-1 (5.12 μM), during which samples were taken at 72 h.p.i. Created with www.biorender.com. (B) Quantification of SARS-CoV-2 RNA (E copies/μl) from 15 serial passages under two-fold escalating concentrations of remdesivir, with DMSO as a mock. Unpaired parametric t-test with the Holm-Šídák correction for multiple testing was used to compare the means from quadruplicates of one experiment. (C) Dose response curves (parental n = 3, passaged virions n = 4) showing the dose-dependent effect of JQ-1 on the quantities of parental and passaged (P15) SARS-CoV-2 genomic RNA (E copies/μl) in the supernatant at 24 h.p.i. Calu-3 cells were pretreated twice for 48 hours prior to infection with parental and passaged (P15) SARS-CoV-2 virions (MOI = 0.1) for 24 hours under continuous presence of the drug. (D) Quantification of resistance phenotypes of passaged (P15) SARS-CoV-2 virions to JQ-1 treatment. Fold resistance is presented as the IC50 concentrations of passaged (P15) SARS-CoV-2 virions relative to IC50 concentration of the parental virus. (E-F) Dose response curves showing the dose-dependent effect of JQ-1 on the quantities of (E) recombinant SARS-CoV-2 genomic RNA (E copies/μl) and (F) infectious titers (PFU/ml) in the supernatant at 24 h.p.i. Calu-3 cells were pretreated twice for 48 hours prior to infection with indicated recombinant viruses (MOI = 0.1) for 24 hours under continuous presence of the drug. Data originate from triplicates of four independent experiments in genomic RNA (E copie [file ppat.1011657.s006.pdf]
