# Supplementary material for: Pharmacological inhibition of bromodomain and extra-terminal proteins induces an NRF-2-mediated antiviral state that is subverted by SARS-CoV-2 infection
Source: PLoS Pathog. 2023 Sep 25;19(9):e1011657. doi: 10.1371/journal.ppat.1011657 (PMC10629670; doi:10.1371/journal.ppat.1011657)
Supplement: S1 Table — Enrichment of transcription factor binding motifs in accessible ATAC-seq peaks in (A) DMSO-treated infected/DMSO-treated uninfected, (B) JQ-1-treated uninfected/DMSO-treated uninfected, (C) JQ-1-treated infected/DMSO-treated infected and (D) JQ-1-treated infected/JQ-1-treated uninfected contrasts. The motif search was conducted using the DREME algorithm to annotate the motifs to the known transcription factor families. The height of the letter represents the frequency of each base in the motif. (PDF) [file ppat.1011657.s007.pdf]

A

DMSO Infected/DMSO Uninfected

TFs	Motif	p-value
IRF8, IRF9, IRF4, STAT1::STAT2, IRF7, IRF3, IRF1, IRF2, IRF5	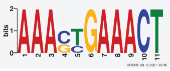	1.8×10^{-20}
RELA, NFKB2, NFKB1, REL	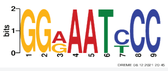	6.2×10^{-19}
RELA	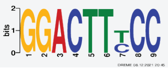	1.2×10^{-07}
EWSR1-FLI1	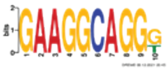	3.1×10^{-07}

B

JQ-1 Uninfected/DMSO Uninfected

TFs	Motif	p-value
FOS, FOSL2, JUNB, FOSB::JUNB, JUN(var.2), FOSL1, FOSL2::JUNB, FOSL2::JUND, FOS::JUNB, JUND, BATF::JUN, FOS::JUN, FOSL2::JUN, JDP2, BACH2, FOS::JUND, FOSL1::JUN, NFE2, Bach1::Mafk, FOSL1::JUNB, MAF::NFE2, FOSL1::JUND, MAFK, JUN::JUNB, Nfe2l2, MAFF, MAFG	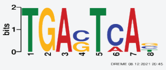	1.2×10^{-288}
NFYB, NFYA	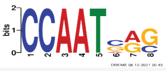	1.4×10^{-224}
Gata4, Gata1, GATA2, GATA3, GATA6, GATA5, Mecom, GATA1::TAL1	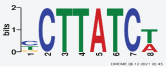	8.6×10^{-100}
Gabpa, ELK4, ELF4, ELK1, ZBTB7A, EHF, ELF1, ELF3, ELK3, ETV6, ETV1, FEV, ETV4, ETV5, FLI1, ERF, ELF5, ETS1, ERG, ETV3, ETV2, SPI1, SPIC, SPDEF	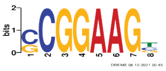	3×10^{-83}
SP2, SP1, KLF5, SP4, KLF14	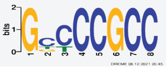	2.7×10^{-75}
TEAD3, TEAD2, TEAD1, TEAD4	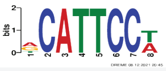	2.7×10^{-68}
RUNX1, RUNX3	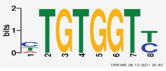	5.6×10^{-57}
NR1H2::RXRA, Rxra, RXRB, Sox2, RXRG, Nr2f6, Sox3	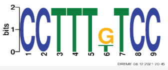	3.1×10^{-11}
Gabpa, ELF3, ETV6, ELF4, ELK1, ELK4, ELF1, ZBTB7A, EHF, ELK3, ERF, ETV3, FEV, FLI1	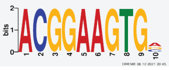	6.9×10^{-10}
RELA, REL, NFKB1, NFKB2	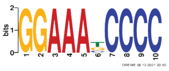	5.7×10^{-09}

C

JQ-1 Infected/DMSO Infected

TFs	Motif	p-value
FOSL2::JUNB, FOSB::JUNB, JUN(var.2), JUNB, FOSL2, FOSL2::JUND, FOS::JUNB, FOS, JUND, FOS::JUN, FOSL1, FOSL2::JUN, BATF::JUN, JDP2, FOS::JUND, FOSL1::JUN, MAF::NFE2, FOSL1::JUNB, NFE2, Bach1::Mafk, Nfe2l2, BACH2, FOSL1::JUND, JUN::JUNB, MAFK, MAFF, MAFG	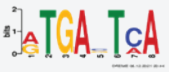	0
Gata4, Gata1, GATA2, GATA3, GATA6, GATA5, Mecom	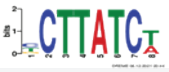	$8.5x10^{-232}$
SP1, SP2, KLF5, KLF16, KLF14, E2F4, E2F6, ZNF740, SP4, KLF13	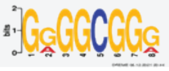	$3.8x10^{-203}$
TEAD3, TEAD2, TEAD1, TEAD4	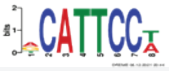	$3.8x10^{-159}$
Gabpa, ELK4, ELF4, ELK1, EHF, ELF3, ELF1, ELK3, ZBTB7A, ETV6, ETV1, FEV, ETV4, FLI1, ERF, ETV5, ELF5, ETS1, ERG, ETV3, ETV2, SPI1, SPDEF, SPIC	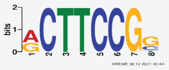	$1.9x10^{-155}$
RUNX1, RUNX3, RUNX2	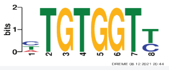	$7x10^{-137}$
NFYA, NFYB, Dux	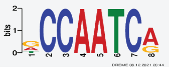	$5.9x10^{-132}$
NRF1	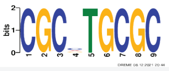	$3.3x10^{-122}$
ONECUT3	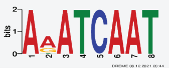	$5.9x10^{-55}$
SP1, SP4, SP2, KLF5, KLF14, KLF16, KLF13, Klf12, Klf1, SP3, SP8, ZNF740	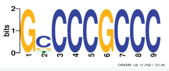	$3.2x10^{-45}$
ZBTB33, ZBED1	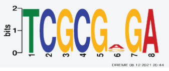	$6.8x10^{-45}$
YY1, E2F2	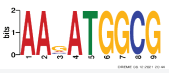	$5.9x10^{-38}$
ETV2, FLI1, ELK4, ETS1, ERG, Gabpa, ELK1, E2F7, FEV, ELK3, ERF, ELF1, ETV1, ETV4, ELF4	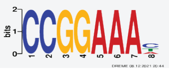	$4x10^{-33}$
NR1H2::RXRA, Rxra, RXRB, NR4A1, RXRG, Rarb, Rarg, Nr2f6, Nr2f6(var.2), RARA, NR2F2, Rarb(var.2), ESRRB, Nr2e1, RARA(var.2), Esrrg, Esrra, Rarg(var.2), NR2F1	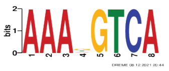	$4.2x10^{-22}$
HOXC12, HOXD12, HOXC10, HOXC11, HOXD11	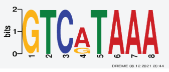	$5.6x10^{-21}$
KLF13, SP4, KLF14, KLF16, Klf12, KLF5, SP3, SP8, Klf1, KLF9, SP1, SP2, Ahr::Arnt	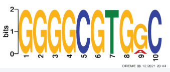	$2.3x10^{-18}$
BHLHE41, USF2, SREBF2(var.2), Arntl, TFE3, Srebf1(var.2), MLX, USF1, BHLHE40, Arnt, MLXIPL, HES5, HEY2, TFEB, MITF, Id2, HES7, HEY1, Creb3l2, Mlxip, MAX, MNT, TFEC, MYCN, Npas2, MXI1, MYC, CLOCK, MAX::MYC, ARNT::HIF1A, CREB3, CREB3L1, PAX9, NR2F1, XBP1, PAX5, Atf1	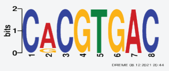	$4.6x10^{-16}$
ELF1, SPDEF, ELF4, ELF3, EHF	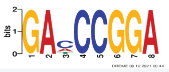	$5.2x10^{-12}$
ZNF143, ZNF410	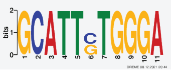	$3.6x10^{-11}$

D

JQ-1 Infected/JQ-1 Uninfected

TFs	Motif	p-value
BATF::JUN, FOS, JDP2, FOSL2::JUNB, NFE2, FOSB::JUNB, JUND, FOSL2::JUND, FOS::JUNB, JUN(var.2), JUNB, FOS::JUN, FOSL2, FOSL1, FOSL2::JUN, FOS::JUND, FOSL1::JUN, MAF::NFE2, FOSL1::JUNB, Nfe2l2, Bach1::Mafk, BACH2, FOSL1::JUND, JUN::JUNB, MAFK, MAFF	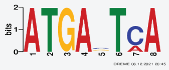	$1.2x10^{-135}$
SP1, SP2	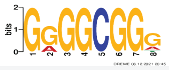	$3.8x10^{-93}$
NRF1	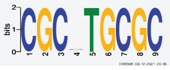	$3.3x10^{-55}$
ZBED1, ZBTB33	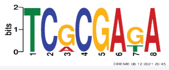	$9.3x10^{-32}$
SP4, SP1, KLF5, SP2, KLF16, Klf12, KLF14, KLF13, SP3, SP8, Klf1	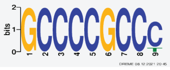	$2.2x10^{-27}$
ELK4, Gabpa, ELK1, FLI1, ELF1, ELK3, ERF, ELF4, FEV, ETS1, ERG, ETV1, EHF, ELF3, ZBTB7A, ETV4, ETV2, ETV3, ETV5, ELF5, SPDEF, ETV6		$1.7x10^{-33}$
FOSB::JUNB, FOSL2::JUNB, FOSL2::JUND, FOS::JUNB, JDP2, JUND, FOS::JUN, FOS, JUN(var.2), FOSL1, JUNB, FOSL2::JUN, FOSL2, NFE2, FOS::JUND, FOSL1::JUN, FOSL1::JUNB, BATF::JUN, Bach1::Mafk, FOSL1::JUND, JUN::JUNB	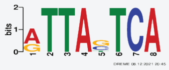	$2.6x10^{-24}$
YY1, E2F2	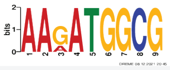	$7.8x10^{-19}$
BATF3, JDP2(var.2), ATF7, JUND(var.2), CREB1, JUN, Creb5, FOSL2::JUNB(var.2), FOSB::JUN, FOSL2::JUND(var.2), FOSL2::JUN(var.2), FOSB::JUNB(var.2), JUN::JUNB(var.2), FOSL1::JUN(var.2), FOS::JUN(var.2), JUNB(var.2), Crem	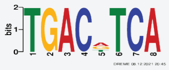	$1.1x10^{-14}$
Rarb, Rarg, RARA, Nr2f6(var.2), NR4A1	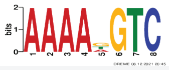	$4x10^{-14}$
Gata1, GATA2, Gata4, GATA6	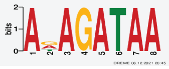	$8.4x10^{-12}$
NRF1	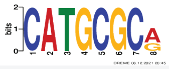	$9x10^{-12}$
BHLHE41, USF2, SREBF2(var.2), Srebf1(var.2), Arntl, MLX, USF1, BHLHE40, Arnt, TFE3, MLXIPL, HES5, MITF, HES7, Creb3l2, HEY2, TFEB, Id2, HEY1, Mlxip, MNT, MAX, Npas2, TFEC, MXI1, CLOCK, MYCN, MYC, CREB3, MAX::MYC, XBP1, ARNT::HIF1A, CREB3L1, PAX9, NR2F1, HIF1A, Atf1, PAX5	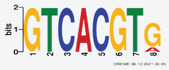	$1.1x10^{-11}$
Dux, PBX1	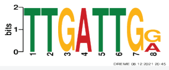	$2.2x10^{-09}$
ELF1, ELF4, ELF3, SPDEF, EHF	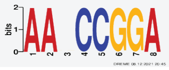	$1.1x10^{-10}$
E2F7	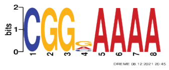	$1.4x10^{-08}$
