# Supplementary material for: Pharmacological inhibition of bromodomain and extra-terminal proteins induces an NRF-2-mediated antiviral state that is subverted by SARS-CoV-2 infection
Source: PLoS Pathog. 2023 Sep 25;19(9):e1011657. doi: 10.1371/journal.ppat.1011657 (PMC10629670; doi:10.1371/journal.ppat.1011657)
Supplement: S2 Table — Codons mapped to SARS-CoV-2 ORF6 between positions 27217–27219 in the genome of (A) input SARS-CoV-2 and (B) serially passaged (P15) SARS-CoV-2 virions under increasing concentrations of DMSO and JQ-1 in Calu-3 cells. The stop codon is marked with an asterisk. (PDF) [file ppat.1011657.s008.pdf]

A

CODONS	FREQUENCY (%)	
	**DMSO**	**JQ-1**
GAC	98.313	5.707
TAA*	1.585	94.254
TAC	0	0.010
GTC	0.010	0
AAC	0	0
GAT	0.031	0
GGC	0.041	0
GAG	0.020	0
CAC	0	0
GAA	0	0
TAT	0	0
CAA	0	0.010
AAT	0	0.010
AAA	0	0.010

B

CODONS	FREQUENCY (%)
GAC	99.455
TAA*	0.182
TAC	0.182
AAC	0.045
GAT	0.091
GAG	0.045
